# Environmentally Friendly Methods for Flavonoid Extraction from Plant Material: Impact of Their Operating Conditions on Yield and Antioxidant Properties

**DOI:** 10.1155/2020/6792069

**Published:** 2020-08-28

**Authors:** Sara Luisa Rodríguez De Luna, R. E. Ramírez-Garza, Sergio O. Serna Saldívar

**Affiliations:** ^1^Tecnologico de Monterrey, Centro de Biotecnología FEMSA, Escuela de Ingeniería y Ciencias, Av. Eugenio Garza Sada 2501 Sur, Monterrey, Nuevo León C.P. 64849, Mexico; ^2^Tecnologico de Monterrey, Tecnologías Sostenibles y Civil, Escuela de Ingeniería y Ciencias, Av. Eugenio Garza Sada 2501 Sur, Monterrey, Nuevo León C.P. 64849, Mexico

## Abstract

The flavonoids are compounds synthesized by plants, and they have properties such as antioxidant, anticancer, anti-inflammatory, and antibacterial, among others. One of the most important bioactive properties of flavonoids is their antioxidant effect. Synthetic antioxidants have side toxic effects whilst natural antioxidants, such as flavonoids from natural sources, have relatively low toxicity. Therefore, it is important to incorporate flavonoids derived from natural sources in several products such as foods, cosmetics, and drugs. For this reason, there is currently a need to extract flavonoids from plant resources. In this review are described the most important parameters involved in the extraction of flavonoids by unconventional methods such as ultrasound, pressurized liquid extraction, mechanochemical, high hydrostatic pressure, supercritical fluid, negative pressure cavitation, intensification of vaporization by decompression to the vacuum, microwave, infrared, pulsed electric field, high-voltage electrical discharges, and enzyme-assisted extraction. There are no unified operation conditions to achieve high yields and purity. Notwithstanding, progress has been achieved in the development of more advanced and environmentally friendly methods of extraction. Although in literature are found important advances, a complete understanding of the extraction process in each of the unconventional techniques is needed to determine the thermodynamic and kinetic mechanisms that govern each of the techniques.

## 1. Introduction

Since ancient times, mankind has benefited from the nutritional and medicinal properties of cereals, legumes, pseudocereals, stems, roots, leaves, fruits, vegetables and their coproducts. Such properties are attributed to phytochemicals associated to these tissues. Phytochemicals are compounds whose biological and pharmacological activities have been recognized over the years. Among the most representative phytochemicals are the flavonoids. The flavonoids are being widely studied due to their abundance and bioactive properties, such as anticancer, anti-inflammatory, antioxidant, antimutagenic, antithrombotic, antiviral, antibacterial, and vasodilator, among others [1, 2]. Flavonoids are secondary metabolites synthesized by plants, providing color and protection from UV light [3, 4], and are considered the most abundant pigments along with chlorophyll and carotenoids. They also help to prevent the oxidation of fats and to protect the vitamins and enzymes in plants. For these applications, mankind has attempted to extract plant flavonoids by creative methods. At the dawn of the intention to extract flavonoids, one of the rudimentary methods (conventional methods) was the heating of plants submerged in water. Ancients were able to obtain compounds of interest by using this simple methodology. Through the years, variations in these methods widened and improved the extraction of phytochemicals using more sophisticated equipment (unconventional methods). This review summarizes the most relevant characteristics of flavonoids, the importance of these compounds for mankind, and conventional and unconventional methods employed to extract these compounds. Moreover, this review presents a general description of equipment and processes, the main parameters and operating conditions utilized during extraction, and the perspectives of these different methods.

## 2. Chemical Structure and Types of Flavonoids

**Figure 1:**
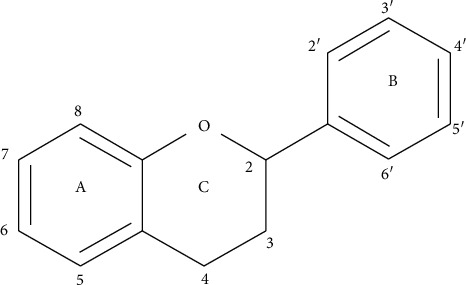
Basic flavonoid structure.

Flavonoids are low molecular weight compounds that share a common skeleton (C6-C3-C6), composed by two rings of phenyls (A and B) linked through a C ring of pyran (heterocyclic). The carbon atoms in rings A and C are numbered from 2 to 8 and those in the ring B are numbered from 2′ to 6′ (Figure 1).

**Figure 2:**
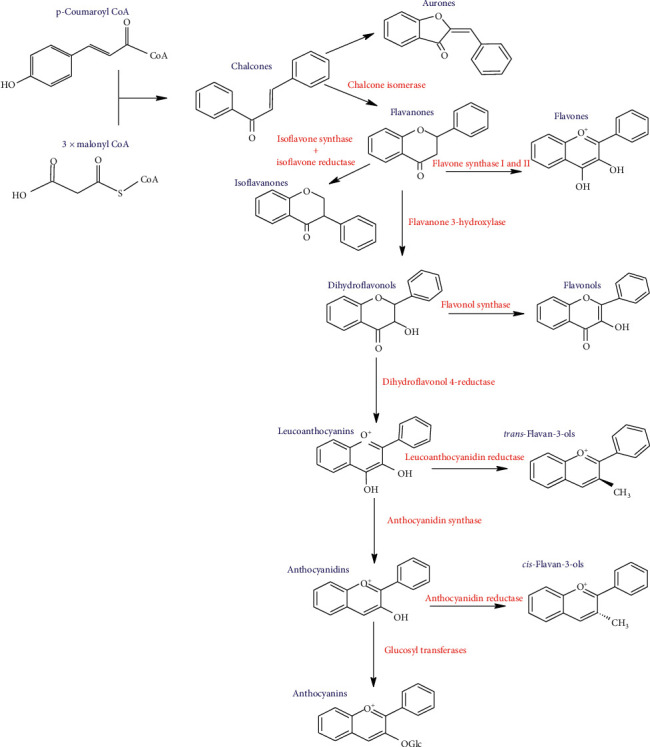
Schematic representation of the flavonoid biosynthetic pathway [3, 5].

This basic structure allows a variety of substitution patterns in the rings. There are some characteristics that are important for the functionality of flavonoids: (a) the presence of the catechol or O-dihydroxy structure in the B ring, (b) a double bond in positions 2 and 3 in ring C, and (c) hydroxyl groups in positions 3 and 5 in C and A rings, respectively. All flavonoids derive their structural skeletons from a biosynthetic reaction that occurs in plant tissues. The synthesis of flavonoids begins with the condensation between three molecules of malonyl-CoA and one molecule of *p*-coumaroyl-CoA to yield an intermediate known as chalcone. Consequently, the chalcones, with the help of some enzymes, act as precursors for the formation of a wide range of flavonoids.  Figure 2 depicts the scheme of the biosynthetic route and the different enzymatic reactions that lead to the formation of several classes of flavonoids.

Flavonoids are classified mainly into 14 groups considering the chemical nature of the molecule and the positions of the substituent groups in rings A, B, and C. The most known types are listed below:

1. Aurones: in the aurones, the pyranic ring of the chalcones is converted to a furan ring.
2. Flavanones: hydroxylated flavanones occur either in the free form or in combination as glycosides in flowers, leaves, fruits, etc. They appear to be of general distribution especially in higher plants.
3. Isoflavanones: they are commonly found in legumes. The 3-phenylchromen-4-one is the base skeleton of the isoflavonoids, which is formed during the biosynthetic pathway, where a benzene ring migrates from position 2 to position 3 of the central ring.
4. Flavones: these flavonoids have the general chemical structure of 2-phenyl-1-benzopyran-4-one. They are found mainly in food products such as honey and grapes.
5. Flavonols: chemically, these flavonoids have as backbone the structure of 3-hydroxyflavone and are present in a wide variety of fruits and vegetables.
6. Anthocyanidins: this type of flavonoid is found in most of wild fruits, especially those that have a purple color. The structural constitution is based on the 2-phenylbenzopyrylium.
7. Anthocyanins: these flavonoids present a similar structure to the anthocyanidins, only that the anthocyanins have a glycosylated part linked to the oxygen substituent of carbon 3. They are also found in red fruits.

Most flavones and flavonols are present as O-glycosides attached preferably on carbon 3 of ring C and less frequently on carbon 7 of ring A. The molecular part of a flavonoid that does not have sugars is known as aglycone. On the other hand, those flavonoids having sugar segments in their structure are known as glycosylated flavonoids. It is important to know in detail the molecular structure of flavonoids for several reasons [5–7]; one of these reasons is that it is known that antioxidant property of flavonoids depends on their hydroxy phenolic groups; other reason is to select the appropriate solvent for the extraction of flavonoids from plant material. Polar flavonoids have affinity for solvents such as aqueous and pure alcohols, whereas nonpolar counterparts such as isoflavones, flavanones, flavones, and flavonols have affinity to solvents such as chloroform, dichloromethane, diethyl ether, and ethyl acetate [8].

Studies on the chemical structure of flavonoids reveal that their solubility is affected due to their capacity to form hydrogen bond with solvents. Glycosylated flavonoids, such as rutin and isoquercetin, show lack of solubility in solvents such as acetone and acetonitrile. The presence or absence of a double bond in C2-C3 of C ring which generates a torsion angle *θ* has generated controversy according to a few studies. This angle involves OC2-C1′-C6′ atoms. Rutin, isoquercitrin, quercetin, and chrysin contain double bond in C2-C3 and angle *θ* of −25°, whereas flavonoids such as naringenin and hesperetin lack this double bond and possess an angle *θ* of approximately 40°. Flavonoids with angle *θ* of 40° are highly soluble in acetonitrile, and therefore solubility is influenced by the angular torsions [8, 9].

## 3. Natural Sources of Flavonoids

The main sources of flavonoids are fruits, vegetables, seeds, and flowers. They are also found in beer, wine, and green and black tea, which are consumed by humans in the typical diet [6]. Another source of flavonoids is agroindustrial wastes [10]. Flavonoids are mainly located in the leaves and flowers; therefore, these parts are the most used for extraction. On the other hand, bark and fresh leaves are less appropriate sources and are more difficult to handle for the extraction of flavonoids because these tissues contain waxes and resins. Particularly, the fresh leaves contain considerable amounts of chlorophyll. All these impurities are commonly eliminated with the application of some pretreatments. The seeds contain large amounts of oil; therefore, polar flavonoids are the easiest to extract with solvents of similar polarity [7]. Below are shown different types of natural sources commonly employed for flavonoid extraction.

### 3.1. Flowers

Flowers display a large variety of colors; this is because they contain some coloring compounds, essential oils, terpenes, carotenoids, organic acids, and flavonoids. The flowers are probably the most convenient source for the extraction of flavonoids because they contain small amounts of impurities, and therefore flavonoids are easier to remove. The most common impurities associated to flowers are mucilages, waxes, and carotenoids; the latter can be removed with aqueous solutions of alcohol and petroleum ether [7, 11–13].

### 3.2. Fruit and Vegetable Peels

Recently, the amount of wastes, particularly from fruits and vegetables, has increased, in response to the rapid growth of the food industry and food consumption. The accumulation of these leftovers causes a great environmental problem especially in urban areas. To counteract these environmental problems, recycling programs have been designed to reuse the husks, seeds, and pulp of some fruits and vegetables. The intention to use fruit residues has been gaining popularity, mainly in the husks, since it has been shown that they contain large quantities of health-promoting polyphenolic compounds, such as flavonoids. In some fruits, the peel represents 30% of the total weight; therefore, peels of fruits such as plum, mango, watermelon, orange, grapefruit, and tangerine have served as natural sources for the extraction of important flavonoids that are known to exert positivity in human health, environment, and economy of communities [14–16].

### 3.3. Seeds

The seeds, in addition to flavonoids, contain fatty acids and essential oils. The isolation of flavonoids, or compounds insoluble in oil, is relatively simple, since the oil can be effectively extracted with petroleum ether. In this way, flavonoids have been recovered from seeds of *Gloriosa superba* [17], *Paullinia cupana* [18], *Ziziphus lotus* [19], and *Salvia hispanica* L. [20], among others.

### 3.4. Leaves

The leaves contain a large amount of chlorophyll, and when they are thick and fleshy, they present large quantities of waxes and resins. In recent years, some researchers have worked in the extraction of flavonoids from some leaves, for example, from green tea (*Camellia sinensis*) [21], mandarin (*Citrus deliciosa* Tenore) [22], and *Cecropia* species [23].

### 3.5. Barks

There is a high number of polyphenols present in the bark of some species of pines and other trees. However, the recovery of these compounds is difficult due to the content of cellulose, hemicellulose, lignin, and fatty compounds associated to cell walls. Therefore, researchers have focused their work on improving the flavonoid recovery methods of some barks, especially when highly lignified barks are wasted from the forestry industry. Recent work has mentioned the extraction of flavonoids from the bark of *Pinus radiata* [24], chestnuts species [25], *Caesalpinia ferrea* C. [26], *Pinus halepensis* [27], *Quercus laurina*, *Quercus crassifolia*, and *Quercus scytophylla* [28].

### 3.6. Roots

It is well known that the roots of some plants have antioxidant and antibacterial properties. For this reason, they are considered as functional and nutraceutical foods. The roots also contain small amounts of resins and essential oils, which can be easily removed with petroleum ether and an aqueous solution of potassium, respectively [7]. *Withania somnifera* [29], *Scutellaria baicalensis* Georgi [30], *Pueraria lobatae* [31], and *Asparagus officinalis* [32] are some examples of roots that have been used as natural sources for the obtention of flavonoids.

### 3.7. Stems

Globally, the agricultural industry produces a large amount of biomass, which includes seeds, cereal straw, and plant stems. Every year, agricultural residues accumulate and most of them are discarded, and only a small amount is used for animal feed or alternatively for energy production. Therefore, many researchers have been interested in these residues, since stems have an important biological activity. Most of these stems are composed of lignin, polysaccharides, and polyphenols. Some stems studied for flavonoid extraction are *Aronia melanocarpa* [33], red pepper [34], *Angelica keiskei* [35], *Flammulina velutipes*, and *Hypsizygus tessellatus* [36]. The use of these diminishes environmental pollution, and these are natural sources of bioactive compounds bringing socioeconomic benefits to local regions.

### 3.8. Grains

Agroindustrial wastes or residues originated from the production of corn, wheat, rice, sorghum, barley, and oats are generated annually in large quantities. These wastes could be a promising source of compounds with biological activity, especially with antioxidant characteristics. The residues of grains have a very complex chemical constitution, and consequently, the extraction of flavonoids is a complicated process. Some samples require a previous preparation to the extraction process, since they are complex natural matrixes, and even factors like humidity and temperature during grain storage can affect the properties of flavonoids. In this context, there are works on the extraction of flavonoids from different grains, for example, from brewer spent grains [10], soybean [37], *Sorghum bicolor* L. [38], oats, corn, wheat, and rice [39, 40].

### 3.9. Fruit and Vegetable Pulps

A frequent consumption of fruits and vegetables among the population is associated with a lower risk of chronic degenerative diseases due to the intake of bioactive compounds present in their pulps. These tissues contain different types of flavonoids. Currently, there are several investigations which focus on studying and developing improved methods for the extraction, purification, and identification of these phytochemicals. The fruits that have been recently studied for this purpose are Sanhua plum [41], watermelon (*Citrullus lanatus*) [42], mandarin (*Citrus reticulata* Blanco) [43], *Opuntia* species [44], *Cucumis metuliferus* [45], and *Euphoria longana* Lam. [46].

## 4. Importance of Flavonoids in Humans and Animals

The flavonoids cannot be synthesized by humans and animals. However, they are an integral part of mammalian diets, and their daily intake varies from 50 to 800 mg [47, 48]. Fruits and vegetables are the main dietary sources of flavonoids for humans, along with tea and wine, and their ingestion typically produces no or very little toxicity [6, 48, 49].

The flavonoids are considered potential natural antioxidants, due to their ability to eliminate free radicals and inhibit their formation. In addition, chelation of metal ions to inhibit lipid peroxidation is another characteristic of flavonoids [50]. In this way, flavonoids could be used to treat some pathophysiological conditions that involve free radicals, such as cardiovascular and neurodegenerative diseases [51]. The antioxidant activity of flavonoids is mainly due to the presence of phenolic rings and free hydroxyl groups in their chemical structure. These free hydroxyl groups can donate hydrogen and thus avoid an oxidation process [52].

Some synthetic antioxidants are provided to humans by drugs, and there have been reports of their toxic effects. For this reason, it has increased the interest in the search for natural antioxidants of plant origin. The use of drugs made with natural antioxidants has been shown to be relatively nontoxic, safe, and free from serious side effects [50]. The incorporation of antioxidants from natural origin into the food, cosmetic, and pharmaceutical industry implies the study of extraction processes suitable for obtaining extracts of high purity and quality. The systematic study of the solubility of flavonoids in different solvents supports the improvement and design of new extraction methodologies [8].

In general, extracts with high purity show greater bioactive capacity. Some authors have reported the degradation of the extracts. Possibly this degradation is due to the prolonged exposure of flavonoids to the high temperatures of the extraction process. Generally, during the extraction processes, the flavonoids are solubilized in organic solvents, and due to their weak acid character, the necessary conditions are created in the chemical medium, for an alcohol dehydration to occur by a mechanism of elimination, in this case, by the polyphenolic structure of the flavonoids.

The flavonoids of the aglycone type are more soluble in polar solvents and the glycosides are more soluble in nonpolar solvents. During the extraction process, those flavonoids exposed to high temperatures and having greater affinity to the solvent will degrade more easily than those with less affinity to the solvent. For this reason, avoiding the degradation of flavonoids ensures the obtention of extracts with greater bioactive potential.

Therefore, it is important to extract the flavonoids from their different plant sources and to study their structure, functionality, metabolism, and bioavailability [6, 48, 49].

## 5. Methods of Extraction of Flavonoids

Currently, two types of extraction methods (solid extraction or leaching) are recognized: (1) *conventional*, which uses simple and low-cost equipment, large amounts of solvent, and extended extraction times working at atmospheric pressure and at relatively higher temperatures, and (2) *unconventional*, which are modern, ecological, and use more expensive and sophisticated equipment that save extraction time and generally can work at higher pressure and temperature values.

Essentially, the general procedure for the obtention of flavonoids has not varied; this broadly consists in the exposition of plant material (hereinafter referred also as solids, plant, or vegetable matrixes) to a solvent allowing the solubilization of phytochemicals.

### 5.1. Conventional Methods of Extraction

Nowadays, the most used conventional procedures for obtention of bioactive compounds are heat reflux, decoction, maceration, infusion, digestion, and percolation. With modernization, an improved conventional maceration and decoction method known as Soxhlet has been extensively employed. Although most of the experiments in conventional methods use water and ethanol as solvents, other environmental hazardous solvents such as methanol, acetone, acetonitrile, ethyl acetate, dichloromethane, hexane, and petroleum ether are used. Temperatures for these methods hardly overpass 100°C; however, in most of these methods, the solvent evaporates usually owing to the fact that the extraction is made in an open vessel. Only heat reflux extraction and Soxhlet methods can recover some of the evaporated solvent. Time of extraction varies in each method, which can be from minutes up to 15 days as in the maceration method. All these inconveniences make the conventional methods disadvantageous compared to unconventional methods. Accordingly, disadvantages are attributed to the thermal degradation of bioactive compounds and the use of stronger and more toxic solvents (nonenvironmentally friendly) [52] which require higher energy consumption. As a result, the use of extracted compounds is more restricted for the food, cosmetic, and pharmaceutical industries [53, 54]. Despite the drawbacks of conventional methods, Soxhlet is considered as a reference method and generally is used for comparison with the more sophisticated methodologies recently developed. In Table 1, a summary of the operating conditions of conventional methods is shown.

### 5.2. Unconventional Extraction Methods

The unconventional extraction methods can be broadly classified in: Mechanical Force Methods (Section 6), Electromagnetic Forces (Section 7), Electrical Forces (Section 8), and Enzymatic methods of extraction for specific biocatalytic reactions (Section 9).

The relatively recent advent of these technologies has placed the unconventional methods as the most recurrent methods, due to global advantages which include:

- Nonthermal effects and prevention of the degradation of thermolabile compounds [84–86]
- Reduction in the amount of solvents
- Reduction of extraction time [87, 88]
- Reduction in energy consumption
- Higher yields and selectivity in the compounds of interest
- Automated and reasonable reproducibility processes
- Environmentally friendly [89]

## 6. Mechanical Force Methods

### 6.1. Ultrasound Extraction

#### 6.1.1. Ultrasound: General Description of Equipment and Process

Ultrasound extraction (UE) is based on the propagation of mechanical waves which produce the formation of cavitation bubbles due to changes in temperature and pressure. When the cavitation bubbles are formed, they collapse during the compression-rarefaction cycles of the mechanical waves. On bursting, bubbles pressure and local temperature can be increased to 100 MPa and 5000 K, respectively, and such conditions induce the plant material to brake facilitating the release of the extracts [90].

**Figure 3:**
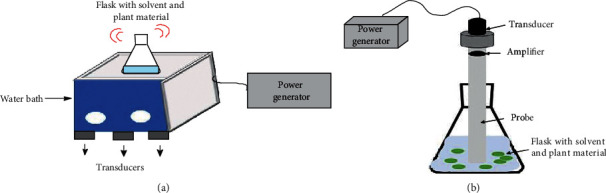
Scheme of ultrasound equipment: (a) bath system; (b) probe system.

The ultrasonic equipment supplies the waves by means of a bath or probe, and they are different in the way they provide the ultrasonic energy. The ultrasonic bath is the most used device, consisting of a stainless-steel tank (Figure 3(a)) that generally operates at frequencies of 40 kHz. On the other hand, the ultrasound system coupled with a probe (Figure 3(b)) generally operates with frequencies of 20 kHz and provides a power 100 times greater than the ultrasonic bath. The ultrasonic probe is immersed directly in the sample vessel, commonly made of glass or stainless steel. Power delivered by the probe is usually higher than bath systems because the energy is supplied through the probe tip; this makes the experiments to be more reproducible than bath equipment. However, probe systems are smaller, and generally, less amounts of plant material are treated.

#### 6.1.2. Main Parameters and Operating Conditions

*(1) Power*. Increasing power usually leads to extract higher amounts of flavonoids, and thus it is an important parameter. Despite this, most of the works in literature do not report the level of power used. The energy from the ultrasonic equipment is transmitted to fluids (liquid solvent and gasses dissolved) and finally converted into kinetic energy when bubbles collapse [91]. Some energy is lost in this transmission from probe or bath to solvent-plant material medium, and therefore measurements of energy absorbed by the plant material should be carried out carefully. In most of the UE works, the power is not adequately related with bubble formation. The importance of this relation is that when bubble collapses, shear forces are created in the liquid in turbulence regimen which ultimately is associated with rupture of cell walls. However, the power should be increased to a point where bubble production does not decrease. In this case, the intensity could play an important role.

*(2) Intensity*. In general, increasing intensities has also contributed to improve the extraction yield, although it might decrease antioxidant properties as well [54]. A clear explanation of the latter has not been revealed; however, it might be associated with the rise of local temperatures at higher intensities, which can provoke chemical degradation. It is important to note that in most of the works by UE, it is found that this method does not affect the antioxidant properties of flavonoids. Notwithstanding, in most investigations, the intensity is not reported (Table 2), and these data are important in order to elucidate their effects in plant matrixes.

*(3) Frequency*. The frequency, another parameter related to energy (and rarely reported), influences the realising of extracts. High frequencies have been found to produce different effects in the solids. These effects can be peeling, erosion, and particle breakdown, although combined effects, such as interparticle collisions, might show fragmentation, sonoporation, capillarity, and detexturization [90]. When the frequency increases, the production of cavitation bubbles also might decrease diminishing the breakdown of plant cell walls. Therefore, careful measurements should be carried out in order to have the optimum value.

*(4) Effects of Particle Size, Temperature*, *and Time of Extraction on Yield*. It is premature to deduce that the effects of ultrasound waves on flavonoid extraction can be enhanced by only varying the frequency because other parameters such as particle size of plant material have also benefits. Jovanović et al. have found that decreasing particle size (≈0.3 mm) of *Thymus serpyllum* L. was highly favorable for flavonoid extraction [57]. The optimum particle size is as small as possible for higher yields, without having technical inconvenience upon management of solids. Also, it has been found that smaller particle sizes and high temperatures increase the extraction of solutes from solids. Generally, an increase in temperature causes the viscosity and surface tension of the liquid solvent to decrease, improving the solubility of bioactive compounds and their mass transfer [98–100]. If solvent is not appropriate to dissolve solute, the most important parameter might be the solvent-solute interaction and not the temperature. A great deal of effort has been made to explain the influence of temperature on the extraction. In this sense, studies have shown that temperature increase has a direct effect by increasing the rate of extraction [101, 102]. Some results reported in the literature have shown a second-order kinetic in which the rate of extraction decreased with increasing temperature for the extraction of polyphenols from *Humulus lupulus* [103]. The increase or decrease in the rate of extraction is still completely unknown and so is the real effect of temperature.

However, it has been observed that temperatures above 75°C promote the degradation of the compounds of interest [90, 104]. Presumably, low temperatures in UE prevent the antioxidant properties to decrease [61, 62, 67]. There are investigations where lower temperatures of UE resulted in higher antioxidant properties compared for instance with accelerated solvent extraction method carried out at higher temperatures; this phenomena was attributed to the influence of extraction technique and solvent nature [105]. On the contrary, other authors have found less antioxidant properties in UE achieved at low temperatures (≈30°C) compared with higher temperatures (≈120°C) of subcritical water extraction method [83]. This might reinforce that there is a strong influence of the method of extraction and the solvent nature where temperature apparently plays a minor role.

A similar situation occurs with time of extraction. In a comparison among *Hypericum formosanum* tissues and *Vitis vinifera* L., apparently a major time of extraction leads to higher yield; however, this not true when comparing with other sources such as *Theobroma cacao* shells (Table 2).

*(5) Effects of Type of Solvent and Moisture on Plant Material and Extract*. Other factors such as selectivity seem to have an important role. If selectivity is influenced by the type of solvent, it is important to assure adequate contact with the plant material. This will allow the extracts to diffuse appropriately. Solvents such as hydroalcoholic mixtures are the most suitable systems for the extraction of phenolic compounds. Normally, mixtures of water with methanol or ethanol are employed. Water acts as a swelling agent for plant material, increasing the contact surface. The use of pure alcohols leads to dehydration and collapse of plant cells, causing the breakdown of the solute-cell wall bond [104, 106]. Therefore, there is a synergistic effect when water-alcohol mixtures are utilized and optimum concentration of alcohol has led to increased flavonoid extraction [107].

Other related parameter with the contact of plant material and solvent is the level of moisture. When a plant has low levels of moisture, the pores of cell walls have a greater capacity to swell which enhances the contact of surface with solvent. As a result, phytochemicals are more easily released.

*(6) Solvent-Solute Ratio*. The solute-solvent ratio is another important factor, especially because it affects the rate of mass transfer. A larger volume of solvent accelerates the diffusion process [108, 109]. The use of large amounts of solvent should lead to a maximum amount of extraction, but even a slight decrease in extraction yield has been noted [109]; although this phenomenon was not clear, probably hydrostatic pressure of large volume of solvent could be playing an important role. It should be pointed out that increasing the solvent volume also increases the cost of postextraction operations, such as filtration and extract recovery; furthermore, the excessive use of solvent also generates higher amounts of waste.

The effect that solid-to-liquid ratio has in yield is owing to the density change of solvent. The density of the solvent might decrease to a point where the velocity of the wave (formed due to shear forces) increases favoring the transfer of energy per unit length [100].

*(7) Amplitude*. When the amplitude of the wave increases, the collapse of the cavitation bubbles is more violent, and consequently, there is a more efficient release of bioactive compounds. Recently, the amplitude has been studied by Orioan et al. for the polyphenol extraction from crude pollen, and they found that it induces a direct effect in cavitation [100].

#### 6.1.3. General Advantages and Disadvantages of UE

One of the advantages of UE is its relatively easy operability, compared to those methods whose equipment requires to generate vacuum or to increase pressure to be operated. In addition, it also has the advantage that it can be used to complement or improve the extraction of other techniques such as supercritical fluids or microwaves. UE is an energy-saving process because normally its time of extraction is short and it still has high selectivity and yields. One of the disadvantages is in the probe system where only small amounts of mass can be treated. However, this issue can be solved by operating in series [90]. The main disadvantage of the bath system is that it has low reproducibility because the water contained in the stainless-steel tank or the glassware attenuates the energy which is not adequately transmitted to the solvent.

### 6.2. Pressurized Liquid/Accelerated Solvent

#### 6.2.1. Pressurized Liquid Extraction: General Description of Equipment and Process

Pressurized liquid extraction (PLE), also known as accelerated solvent extraction, was introduced for the first time in 1995. It is a process that combines high pressures and temperatures with organic solvents above its boiling point [110]. PLE is a method that works with subcritical liquids solvents usually at pressures ranging from 4 to 12 MPa and uses from moderate to high temperatures, ranging from 50 to 300°C (Table 3).

**Figure 4:**
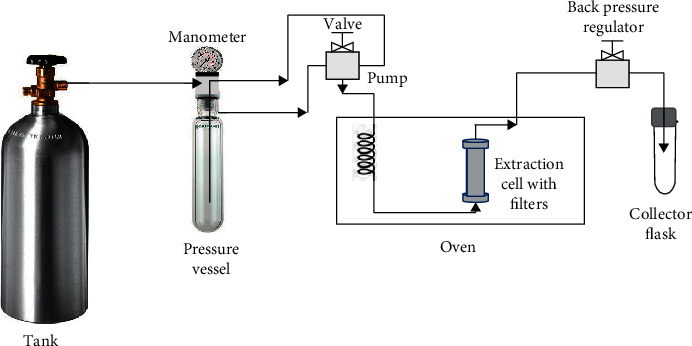
Scheme of pressurized liquid extraction equipment.

There are several models of commercial equipment for the extraction of bioactive compounds by pressurized liquids. Among them is the accelerated solvent extractor ASE200 model (Dionex, Thermo Fisher Scientific, Waltham, MA, USA). This equipment allows the extraction from plant material at high pressure (up to 13 MPa) and high temperature (up to 200°C) [116]. The ASE350 (Dionex, Thermo Fisher Scientific, USA) is a model that is also widely used in PLE extraction, operating with a unique pressure of 10.3 MPa [117, 118].  Figure 4 shows a schematic representation of PLE equipment.

In these systems, a solid sample is enclosed in a cartridge that is filled with an extraction solvent and used to statically extract the sample under elevated temperature and pressure conditions for short time periods (5–10 min). The compressed gas is used to purge the sample extract from the extraction cell into a collector flask (Figure 4) [110].

#### 6.2.2. Main Parameters and Operating Conditions

In the pressurized liquid technique, there are various parameters of extraction which can be modified to improve the extraction yield. The parameters that can be controlled are the type and volume of solvent, pressure, temperature, and time of extraction [110, 116]. Less frequently, some authors have studied the impact of factors such as percentage of dispersant (for the filters) and flushing volume on extraction by pressurized liquids [116].

*(1) Type and Volume of Solvent*. Generally, PLE uses solvents recognized as safe, usually ethanol and/or water. This type of solvents promotes the extraction of bioactive compounds present in the solids, with a reduced amount of volume.

*(2) Pressure and Temperature*. The use of high pressures and temperatures keeps the solvent in a liquid state. Under these conditions, extraction is facilitated. This allows a better contact between the solvent and the bioactive compounds trapped in the pores of the plant material. Normally, this contact does not occur in processes at atmospheric pressure [110].

The use of higher temperatures increases the capacity of solvents to solubilize analytes. An increase in temperature favors the breakdown of the interactions caused by van der Waals forces and hydrogen bonding that occur between bioactive compounds and plant material. Furthermore, it is known that at elevated temperatures, the viscosity of liquid solvent decreases, and this allows a better penetration of the solvent to the pores of the plant matrix, enhancing the extraction. Therefore, at high pressures and temperatures, the solubility and mass transfer properties are enhanced in the extraction [110, 119].

*(3) Time of Extraction*. The efficiency of the method can be improved by increasing the number of extraction cycle [52]. However, there are authors who have found better recovery yields of bioactive compounds using short extraction times [117]. Since there is not a clear trend, the extraction time is a parameter that must be optimized in each experiment.

*(4) Combination of PLE with Other Emerging Methods*. The solubility and mass transfer properties are enhanced using high temperatures and pressures. However, high temperatures can damage thermolabile compounds. Recently, to solve this problem, Viganó et al. decided to combine the PLE technique with ultrasound. PLE assisted by ultrasound increased the yields, resulting in 60% higher of total polyphenols. The authors concluded that high yields have been attributed to the propagation of ultrasound pressure waves through the solvent and resulting cavitation phenomena [119]. Tamkuté et al. recovered polyphenolic compounds from cranberry pomace by consecutive supercritical CO_2_ and PLE, finding a better recovery yield with the combination of both techniques. Moreover, the results of this study create a promising platform for “zero waste” processing of cranberry pomace at the industrial scale [118].

*(5) Perspectives of Pressurized Liquid Extraction*. PLE is a rapid, clean, and environmentally friendly technique for determination of bioactive compounds in plant matrix. For this extraction technique, there are commercial systems, and with these kinds of equipment, the extractions can be programmed and automatically run, which is convenient for quality control. The antioxidant extract of rosemary has been authorized in 2010 by the European Union as food additive E392 (directive No. 2010/69/EU), and since then, these compounds have been extracted at industrial scale by PLE [116, 120]. A disadvantage of the technique could be the high cost of commercial equipment [119].

### 6.3. Mechanochemical

#### 6.3.1. Description of the Stages in the Mechanochemical Extraction

Mechanochemical-assisted extraction (MAE) was used for the first time in 2003 by Korolev et al. [121] for the extraction of triterpenic acids. Since then, this technique has received much attention from the industry due to its zero or little consumption of organic solvents [122].

**Figure 5:**
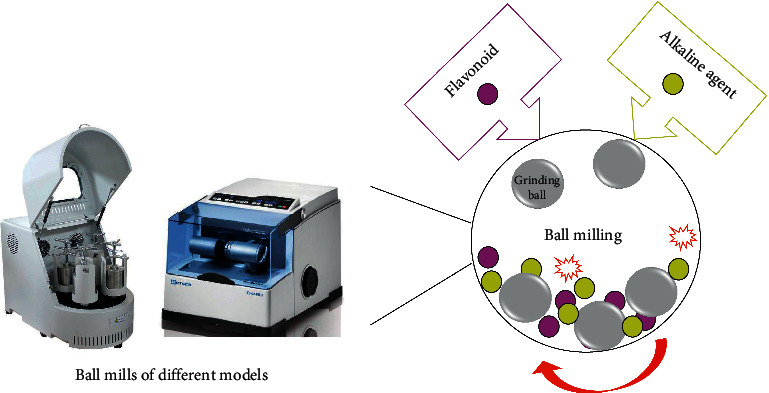
Second step of the extraction process by mechanochemical.

MAE consists of three fundamental steps: (1) pretreatment of the raw material, (2) activation of the raw material by mechanochemical treatment, and (3) subsequent extraction procedures. In the preparation of the sample, the raw plant material is commonly dried and pulverized to achieve a particle size distribution of 0.5 to 2 mm. The material is subsequently stored in dry conditions at room temperature. The second stage occurs in a ball mill, where the plant material is mixed with one of the following reagents: Na_2_CO_3_, NaHCO_3_, NaOH, SiO_2_, hydroxypropyl-*β*-cyclodextrin (HP-*β*-CD), or Na_2_B_4_O_7_.10H_2_O, commonly called alkaline agents (Figure 5) [122, 123].

In this second step, the objective is to change the chemical form of the flavonoids contained in the plant material. Flavonoids are weak acidic molecules, and when they are mixed with an alkaline agent inside the ball mill, a neutralization reaction occurs. The product of the neutralization reaction that is obtained is a flavonoid salt. A salt derived from a flavonoid is chemically more soluble in water than the original flavonoid. The mechanochemical treatment stage allows water to be used as the extraction solvent in the third stage of the process. Finally, the third stage occurs, which is the extraction stage. In this step, the salt derived from the flavonoid is dissolved in water and subsequently is centrifuged; after this, the supernatant is collected and acidified to convert the salt into the original flavonoid, and finally, the extract is concentrated in a rotary evaporator under vacuum to obtain the extract [122, 123].

#### 6.3.2. Main Parameters and Operation Conditions

Several studies have shown that the effectiveness of the MAE depends on (1) particle size of the plant material, (2) type and concentration of the alkaline agent, (3) grinding time, (4) extraction temperature, and (5) acidification pH [52, 122].

*(1) Particle Size*. The reduction in particle size leads to a greater contact surface between the bioactive compounds of the vegetable and the alkaline agent, increasing the probability of the desired chemical reaction. Zhu et al. found that a finer particle size distribution favored the extraction yield of flavonoids and terpene trilactones from ginkgo leaves [124].

*(2) Type and Amount of Alkaline Reagent*. The selection of alkaline reagent depends on the acidic properties of the compounds of interest. Xie et al. reported that NaHCO_3_ was not appropriate for the extraction of flavonoids from bamboo leaves due to alkaline agent weakness, resulting in an uncompleted neutralization [123]. Likewise, kaempferol glycosides were better extracted with a strong alkali (NaOH) in comparison with weak alkalis (Na_2_CO_3_ and NaHCO_3_) [125]. However, if the alkali is too strong, the interest compounds can be adversely affected because of their possible transformation into undesirable products. Zhu et al. found that NaHCO_3_ was the most suitable reagent for the extraction of flavonoids and terpene trilactones from ginkgo leaves and that NaOH and Na_2_CO_3_ destroyed the flavonoids by oxidation generated during grinding [124]. Mixtures of alkaline agents are recurrent alternatives in order to improve extraction yields. For instance, the mixture of NaOH and SiO_2_ reduced the hygroscopic properties of the alkali [126]. Xie et al. mixed Na_2_CO_3_ and Na_2_B_4_O_7_·10H_2_O for routine extraction of *Hibiscus mutabilis* in order to protect the o-phenolic hydroxyl group of rutin and prevent its oxidation [127]. In the same way, Xie et al. added Na_2_B_4_O_7_·10H_2_O with the purpose of avoiding the oxidation of flavonoids from bamboo leaves [123].

The optimum amount of alkaline agent is necessary to extract with the maximum efficiency without forming undesirable products. Xie et al. reported that the yield of flavonoids increased significantly when the amount of Na_2_B_4_O_7_·10H_2_O augmented up to 2% (w/w). However, the reagent concentration greater than 2% (w/w) affected the yield of the compounds of interest [127].

*(3) Grinding Time*. In general, the extraction yield improves with the increase in grinding time. Xie et al. increased the yield of extraction of flavonoids with a time extension from 3 to 10 minutes obtaining yields from 10.75 ± 0.06 mg/mL to 15.33 ± 0.05 mg/mL, respectively [123]. However, excessive grinding time maintained or even decreased the yield due to the formation of conglomerates that provoked partial decomposition. Xie et al. reported that long grinding times negatively affected rutin yields owing to formation and oxidation of conglomerates [127]. Zhu et al. demonstrated that the rigid cell walls of *Camellia oleifera* were destroyed by mechanic forces of grinding. By means of scanning electron microscopy (SEM) studies, they verified that almost no plant cell remained intact [125].

Once the grinding is complete, the mixture of the alkaline agent and plant material must be dissolved by adding the appropriate amount of solvent, which generally is water. The excessive use of solvent makes the manipulation of the extracts difficult.

*(4) Extraction Temperature*. In MAE, room temperature is generally used, approximately 25°C. Xie et al. reported extraction temperatures higher than 25°C were not necessary to improve extraction efficiency of rutin [127]. Moreover, Zhu et al. reported that the extraction yields of flavonoids and terpene trilactones decreased at higher temperatures [124].

*(5) Acidification pH Value*. At the beginning of the third stage of the general process by MAE, there is a dissolution of the flavonoid in the form of salt, and it is important to recover the original flavonoid. To recover the flavonoid, the solution where the salt is dissolved needs to be acidified; therefore, the pH value must be optimized. In general, the solubility of the original flavonoid in water is related to the pH of the solution and its acid dissociation constant. If the pH of the solution is higher than the flavonoid's pKa, the flavonoid will be very soluble in aqueous phases because it will exist in ionized form. Commonly, acetic acid and citric acid are used for these extraction processes [122, 123].

#### 6.3.3. Perspectives of the Mechanochemical Extraction Method

The main advantages of MAE are the use of water as a solvent, the relatively short extraction times, and the easiness of manipulation of the materials during the process. Despite these advantages, there were no kinetic studies to determine the basic data of mass transfer required to further develop the technique. These studies are necessary in order to scale up MAE in industrial settings.

The reaction that occurs in the ball mill between the flavonoid and the alkaline agent needs to be deeply explored. Because this is a solvent-free process, it is of great interest to know the factors that affect it. The scanning electron microscopy (SEM) is a very useful characterization technique to study the morphological changes in reagents processed within the ball mill, and with it, a reaction mechanism could be possibly determined [125, 126].

### 6.4. High Hydrostatic Pressure

#### 6.4.1. High Hydrostatic Pressure: General Description of Equipment and Process

**Figure 6:**
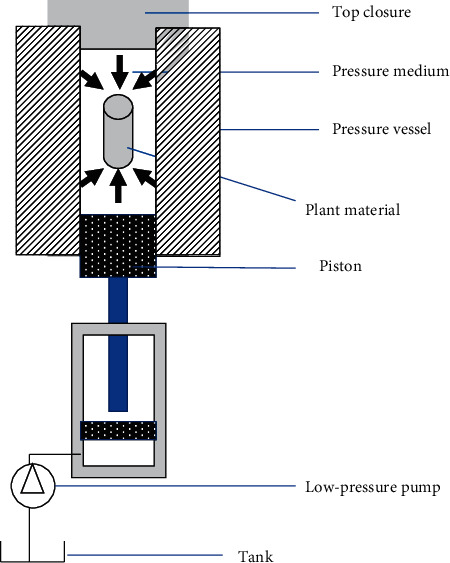
Scheme of high hydrostatic pressure equipment.

High hydrostatic pressure extraction (HHPE) is a novel technique that was first used in 2005 to successfully obtain flavonoids of propolis. The principles of the technique consist of six stages: (1) the dry plant material is pulverized and sifted on 40 or 60 mesh sieves, (2) the appropriate solvent is selected, (3) the pulverized material is mixed with the solvent normally inside a sterile polyethylene bag, which is then purged and sealed, (4) the bag is introduced into a pressure vessel, equipped with valves to release pressure; temperature controller is also installed in vessel, (5) the vessel is pressurized by a fluid, usually water, and once the targeted pressure is reached, the extraction time is programmed, and finally (6) the resulting extract is filtered to remove the solid particles (Figure 6).

#### 6.4.2. Main Parameters and Operation Conditions

The main parameters that affect the extraction by HHPE are the type of solvent, the pressure level, and the extraction time [128, 129].

*(1) Types of Solvents*. The solvent and the target compound polarities should be similar. In the extraction by high hydrostatic pressures, pure solvents or mixtures such as water and polar and nonpolar organic solvents are used. The toxicity of the solvent is an important characteristic, and in general, ethanol is preferred. Normally, a large amount of solvent can dissolve the target compounds in a very effective manner and generate high extraction yields [129].

*(2) Operating Pressure*. This method operates with very high pressures ranging from 100 to 1000 MPa. Generally, the elevated pressure results in a high extraction efficiency. High pressures provoke the rupture of cell wall, increasing the penetration of solvent in the cell. This creates high cell permeability, and consequently, the bioactive compounds are released to the solvent more efficiently [128, 130]. Even though HHPE is almost always carried out at room temperature, the high pressures compress the fluids, increasing the temperature. This is an important aspect to ponder especially when the desired bioactive compounds are heat-sensitive [128, 131].

*(3) Extraction Time*. Briones-Labarca et al. found that flavonoid yield of *Vasconcellea pubescens* seeds improved by increasing extraction time [132]. Another important finding is that the extraction by HHPE also generates greater antioxidant activity of the extracts because HHPE inactivates some degradation enzymes, which can damage the quality of the extracts [132]. Thus, HHPE is an excellent alternative for the extraction of thermolabile flavonoids because the process does not need high temperatures. Despite this advantage, more studies are needed to achieve greater separation and purification of the extracts.

#### 6.4.3. Perspectives in the High Hydrostatic Pressure Extraction

Although HHPE has not been used on an industrial scale, it is a promising method for the extraction of value-added bioactive ingredients in the future. This novel technique needs further research especially in terms of identifying key parameters that affect extraction yields and determination of kinetic models [132].

### 6.5. Supercritical Fluid

#### 6.5.1. General Description of Equipment and Process

**Figure 7:**
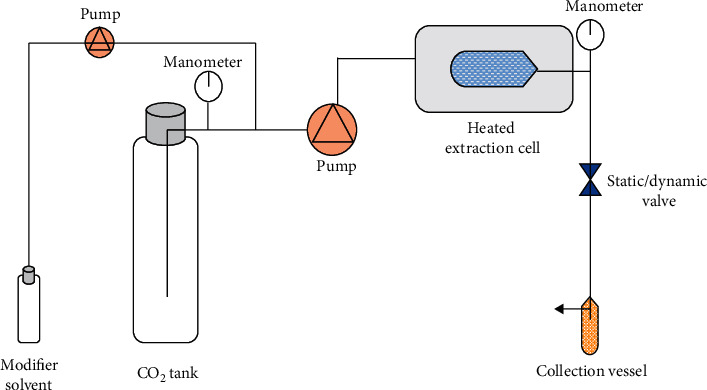
Scheme of supercritical fluid equipment.

The supercritical fluid extraction (SFE) is a method which usually employs CO_2_ as solvent. The use of supercritical fluids (SF) began in the mid-1980s for the extraction of components from plant matrix with great application in the pharmaceutical, cosmetic, and food industries. The SFE has been effective for the extraction of polar compounds such as polyphenols and nonpolar compounds such as lipids and carotenoids. A scheme of this equipment is presented in Figure 7.

#### 6.5.2. Main Parameters and Operating Conditions

*(1) Particle Size*. In this process, the particle size is an important issue to consider. In general, the content of bioactive compounds in the final extract is inversely proportional to the particle size of the plant material. Commonly, it is preferred to work with small particle sizes and to sieve the solids [59, 115]. However, very small particle sizes might cause the agglomeration of the substrate in the compact channels of the equipment, which produces a poor solvent flow and a low recovery yield. On the other hand, the use of larger particles decreases the mass transfer because it hinders the solvent flow velocity. Therefore, in SFE operations, it is relevant to define the suitable mean particle diameter of the plant matrix [133].

*(2) Flow Rate of CO_2_ and the Role of Pressure and Temperature*. In SFE, the flow rate of CO_2_ is applied principally in function of the solute solubility. The flow rate must be sufficiently high to maximize the extraction rate. An optimum value of flow rate is located on the region where both solubility and mass transfer are significant factors [134]. Therefore, increasing flow rate of CO_2_ will not have a strong effect in extraction, if diffusion from the inner cells of plant material is slow; temperature increase is more appropriated if diffusion from the inner cells needs to be faster [135]. An increase in temperature facilitates the diffusion of bioactive compounds to CO_2_ and makes the solute vapor pressure to rise, whereas it reduces both the viscosity and surface tension of the water contained in the plant, allowing a greater penetration of the SF [136]. Temperature must be elevated carefully; its increase provokes the solvent density to decrease, diminishing the solubility of interest compounds [137, 138]. Thus, temperature is the main parameter that influences the selectivity and it is necessary to optimize it in order to increase yield [136, 137]. In recent experiments with beetroot leaves for the extraction of polyphenols, it was found that increasing the pressure increased the extraction kinetics. The researchers attributed this improvement in extraction thanks to the increase in fluid density [139]. However, temperature has a major effect in the extraction than flow rate and pressure.

*(3) Interaction of CO_2_ with Solvent and the Addition of Modifiers*. For the extraction of phenolic compounds, CO_2_ is not the best option, since its polarity is low compared to the polarity of phenolic compounds and their related compounds like flavonoids. This reduces the solubility of flavonoids in CO_2_. Increasing pressure might enhance the solubility of flavonoids in CO_2_ because of the augmentation of density [137]. Therefore, the extraction conditions for the supercritical CO_2_ must be above its critical temperature and pressure (31°C and 74 bar, respectively). As mentioned before, temperature as the main parameter has more effect on solubility, and the increase of pressure does not always have a notable effect on it [140]. If temperature is not to be raised in order to preserve thermolabile compounds, the addition of modifiers to CO_2_ improves the recovery of bioactive compound. The modifiers must fulfill the conditions to be considered as green solvents. Due to their low toxicity, the most used and recommended modifiers are water and ethanol (Table 4) although occasionally methanol and propanol are used. Ethanol addition to CO_2_ has been found to improve the antioxidant properties, and this effect is attributed to the higher amount of phenolic compounds extracted [130, 138, 142]. Nevertheless, the employment of modifiers is not recommended if organic solvent-free extracts are required. In this case, it is preferable to manipulate the pressure and temperature conditions of the process to modify the density of CO_2_ [144]. For this reason, CO_2_ is the solvent par excellence in most of the SFE operations [145, 146].

#### 6.5.3. Perspectives of Supercritical Fluid Extraction

The SFE technique allows the extracts to be cleaner and easier to recover compared to other conventional and unconventional methods because it does not need to concentrate the extracts at the end of the process. It is noteworthy that the CO_2_ used in some SFE processes is largely a by-product of industrial processes, for example, the beer production process, which reduces emissions to the environment [135, 145]. An additional advantage of SFE is the possibility of coupling with gas or liquid chromatographs at the end of the operation, which is normally used for the identification of highly volatile compounds. The SFE method is used for industrial scale, confirming the wide applicability for the process from milligrams in laboratory up to tons in the industry. However, flavonoid extraction by SFE has not been exploited and more research is necessary to understand how the parameters can be modulated in order to increase the yield. The main disadvantage of the method is that the initial cost of the equipment is very high and it requires more expertise to run SFE extraction procedures [135].

### 6.6. Negative Pressure Cavitation Extraction

#### 6.6.1. Negative Pressure Cavitation Extraction: General Description of Equipment and Process

**Figure 8:**
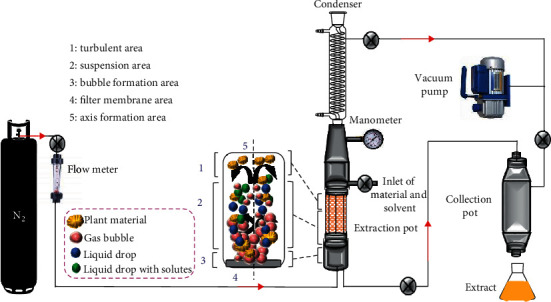
General scheme of a NPCE equipment.

Negative pressure cavitation extraction (NPCE) consists in flowing a gas (usually nitrogen) into a vessel which contains the vegetable matrix and solvent in a vacuum environment. The gas flow and the negative pressure might be controlled by means of control valves [84]. The gas contact with the solvent under vacuum helps to create the cavitation process which produces turbulence and violent movements of the solvent and solids.  Figure 8 shows a scheme of a general equipment process of NPCE. As a result, cells are damaged due to the collision of solid and bubbles, and this mechanism improves the mass transfer of the flavonoids from the inside of the cells to the solvent [85, 147].

#### 6.6.2. Main Parameters and Operating Conditions

Generally, in the NPCE experiments, several parameters are optimized including the grain or solid size, type of solvent, temperature, negative pressure, solvent-to-solid ratio, and time of extraction [147]. These parameters have been compared with other techniques such as UE or heat reflux extraction [148].

*(1) Particle Size*. Small particle sizes obtained when the dry solids are ground to pass through the range of 80 to 100 mesh are reported to favor the extraction of flavonoids [147]. Although it is likely that high gas flow leads to expulsion of solids from vessel, no investigations were found where they reported technical problems with the management of small particle sizes.

*(2) Solvents' Interaction with Solids*. Organic solvents such as methanol or ethanol might reach inner cavities or pores of the vegetable matrix and extract compounds by solubilization [85, 149]. Notwithstanding, during the NPCE process, ethanol has been found to evaporate, lowering extraction yields [148]. In order to overcome this obstacle, solvent alternatives have been proposed such as the use of ionic liquid (IL) or deep eutectic solvent (DES). ILs are composed of a mixture of organic cations with organic or inorganic anions. Thus, their physicochemical properties differ from organic solvents especially in terms of evaporation, viscosity, the way they interact with both polar and nonpolar molecules, and their miscibility ability with organic or inorganic solvents [150]. DESs are similar to ILs in their properties of interaction with polar and nonpolar molecules, which increase extraction. DESs have the advantages of comparatively lower melting point and lower cost and they are more environmentally friendly than ILs [151].

*(3) Temperature and Pressure Effects*. Extraction of flavonoids is affected mainly as a function of the solvent temperature. Temperatures higher than 80°C negatively affect extraction probably due to degradation. Thus, extraction yields are found to diminish by increasing temperatures [148]. Likewise, pressures around −0.05 MPa increases the degradation of extracted flavonoids. However, pressures below −0.05 MPa have also been found to improve mass transfer due to the more violent interaction during process [147, 149]. A counterexplanation is given by other researchers, who documented that pressures lower than −0.05 MPa provoked the nitrogen to decrease; this diminished the bubble formation and cavitation which consequently lowered the extraction efficiency [84]. Negative pressure has been ascribed therefore as an important parameter which is usually tested to find its optimum value.

*(4) Liquid-to-Solid Ratio and Time of Extraction Effects*. Other parameters to control are the liquid-to-solid ratio and time of extraction. Increasing the liquid-to-solid ratio normally leads to improve the extraction yield. Generally, extreme liquid-to-solid ratios do not favor the cavitation process, negatively affecting extraction yields [85, 147, 148]. Normally, it has been found that increasing time of extraction produces higher yields. Notwithstanding, degradation of flavonoids might occur when time extends beyond 30 minutes [85]. The reason for this was not explained; however, collisions may increase temperature, finally affecting the properties of extracts.

#### 6.6.3. Perspectives of Negative Pressure Cavitation Extraction Process

The optimization of processing parameters has been performed with hybrid or synergistic methodologies along with NPCE. In this way, NPCE has been improved by combining with microwave and ultrasound-assisted extractions. For instance, the synergistic operation of NPCE and ultrasound (U-NPCE) helped to increase the extraction yield of total flavonoids due to higher rupture of cell wall [69, 149]. These combinations of techniques with NPCE are being researched to improve extraction yield and to optimize parameters in order to scale up processes with industrial applications.

### 6.7. Intensification of Vaporization by Decompression to the Vacuum

There is a method that has been used for the recovery of defatted, dehydrated, and distorted kernel seeds, known as intensification of vaporization by decompression to the vacuum (IVDV). Specifically, this method is used, for example, when some seeds that will be consumed as food are dehydrated for their good conservation; however, seeds can become hardened to be edible, and therefore an IVDV pretreatment is necessary.

The IVDV process is a mechanical method based on the rapid pressurization of water vapor through a steam generation system. The generated pressure is close to 1.5 MPa, and this pressure is reached in 1 s or less. The vegetable matrix is exposed to this pressure and is subsequently depressurized in a vacuum chamber. During this operation, pores are produced but substances such as polyphenols can also be lost. In fact, one of the parameters that is measured in IVDV is how much the process affects the loss of polyphenols. It has been found that under certain values of pressure and moisture content, massive loss of polyphenols can be avoided [152]. In addition, the seed texture, far from being hard, becomes processable. For this reason, it is seen as a potential technique for the pretreatment of plant material for flavonoid extraction.

## 7. Electromagnetic Force Methods

### 7.1. Microwave

#### 7.1.1. Microwave: General Description of Equipment and Process

Nowadays, besides their wide use as kitchen appliance, microwave ovens have gained popularity in the scientific research community. The microwave-assisted extraction (MWAE) technology efficiently extracts compounds of interest associated to plant materials and is one of the preferred methods.

**Figure 9:**
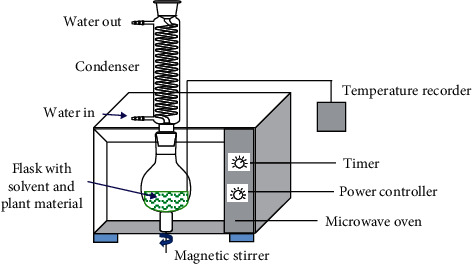
Scheme of a general MWAE equipment.

Although special MW equipment for laboratory and scientific research applications is already commercially available, some laboratories still use MW ovens bought as kitchen appliance. However, this kitchen equipment is usually modified and adapted for the conditions needed. Some adequations of kitchen MW are the installation of a condenser, placed outside the oven to recover the solvent lost and the instalation of a temperature controller (Figure 9). The MW power source is a default device and most of the equipment have an adequate power controller. Commercial MW equipment, specially designed for research laboratories, offers more versatility in power, temperature and time, than kitchen equipment. The extraction process for compounds of interest follows the same operation independently of the type of MW equipment.

#### 7.1.2. Main Parameters and Operating Conditions

*(1) Effects of Particle Size and Different Sources*. In the microwave-assisted extraction (MWAE), the plant material is normally first dried, powdered, sieved, and stored at refrigeration temperatures (4–10°C). Most investigations only focus on a single particle size. The particle size and mass used depend on the type of solids, and these usually range from 60 *μ*m up to 70 mm and from 1 g to 0.5 kg. In the literature reviewed, there is no clear correlation among particle size and yield of flavonoids. For sources of flavonoids such as *Terminalia chebula*, a very low size of particle was used (64 *μ*m), obtaining a yield of 23.35 mg of quercetin equivalent per gram of solid [153], whilst for pitaya fruit, a particle size below 900 *μ*m and a yield of 1.51 mg of betacyanins per gram of solid were obtained [154]. Although the sources of flavonoids are different, a decrease in particle size does not imply higher yield of flavonoids, and MW effect in yield may be roughly independent of particle size. This is a probable reason that variation in particle size is one of the parameters less weighed in MWAE and generally only one kind of mesh is used. MWAE experiments are achieved mostly with fresh biomass, and only a few experiments use storage biomass. Storage effects are also an issue never accounted in evaluation of extraction in MWAE.

*(2) Power Effects on Yield*. After having a uniform particle size, extraction usually takes place in Erlenmeyer or volumetric flasks. The solids are placed into the flask and a solvent is poured depending on the sample weight. During this process, the sample and solvent are exposed to MW radiation. Power of MW is one of the most varied parameters because it is closely related to extraction yield. The range of power in MWAE varies from 100 up to 900 W (Table 5). The MW radiation interacts with the sample especially at the beginning of the extraction, whereas the interaction between radiation and solvent produces a thermal effect, heating the solvent. Heating is a function of the MW frequency and power. Thermal effects are related with frequencies and dielectric properties of solvents. Thus, solvents or other materials are prone to be polarized by their interaction with microwave irradiation. This polarization might be as electronic, dipolar, ionic, or interfacial modes [163]. Solvents like water molecules, which present a dipolar moment, might be trying to align the electric field. Due to an alternating electric field, dipolar polarization occurs out of phase, leading to a dissipation of energy (heat effect) [163]. This is an important issue since temperature increase has a kinetic effect, improving the rate of extraction, and antioxidant properties are conserved at moderate temperatures [153, 164, 165]. Although MW power is one of the principal parameters, its effects on the yield is not clear. For instance, 600 W used for pitaya fruit led to lower yield compared with 560 W and 416 W used for epiphytic ferns and *Vernonia amygdalina* leaves, respectively [82, 154, 165]. Moreover, optimum values of MW power reduce thermal degradation and avoid the loss of antioxidant properties of flavonoids [82].

*(3) Solvents' Effects*. The solvent most used in MWAE is ethanol with variable concentrations followed by water. The third most used solvent is methanol and chloroform is rarely used for this method [166, 167]. Solvent concentration might present optimum values as was found in *Moringa oleifera* leaves [114] and *Achillea millefolium* [168]. Similar concentrations in flavonoid extraction from *Achillea millefolium* and *Arbutus unedo* L. and *Satureja macrostema* produce comparable yields; when concentration of ethanol is lower, as in *Oryza sativa* cv. or *Periploca forrestii*, yields decrease considerably (Table 5). Therefore, higher concentration of ethanol generally can be related with high extraction yield. Solvents such as water extracted higher amounts of total phenolic compounds compared with ethanol, from epiphytic ferns. This was attributed to the presence of carbonyls and organic acids which were more rapidly dissolved in water and associated with a polarization provoked by MW [165]. This phenomenon has been explained in terms of the relative polarity which enhances the contact with solvent such as the mixtures of methanol/water [169]. Also, methanol has been found to be more adequate for quercetin extraction compared with ethanol solvent in *Pithecellobium dulce* [170]. Notwithstanding, the effects on yield of different solvents applied to the same solids have been barely examined. There is no clear tendency between time of extraction and the type solvent used (Table 5); moreover, solvent effects along with MW power on plant material and flavonoids are not well completely understood. A narrow window of wavelength from MW source is probably needed to observe their specific effects.

*(4) Solvent-Free MWAE*. Solvent-free MWAE has also been conducted in a process known as vacuum microwave hydrodiffusion gravity extraction (VMHG) [171, 172]. A variation of this method might be without vacuum such as microwave hydrodiffusion and gravity (MHG). This method uses the MW as a pretreatment, and after the solids have been exposed to MW radiation, the extraction is achieved by other methods. For instance, the MHG has been recently applied as pretreatment for the extraction of bioactive compounds from *Camellia sinensis* leaves by ultrasound. The effectiveness of MHG depends on the extent of damage in cell wall, and in this study, power higher than 100 W was found to be more effective. Interestingly, microwave pretreatment did not reduce the antioxidant properties such as ultrasonic process of extraction in which a time longer than 15 minutes resulted in detriment of the antioxidant properties [173].

#### 7.1.3. Perspectives of the Microwave-Assisted Extraction Method

MWAE presents clear advantages compared with conventional extraction methods especially in terms of using nontoxic solvents or even the possibility of performing extractions without solvent. Furthermore, MWAE allows the design of different schemes to vary the selectivity for specific substances. For example, MWAE has effectively been used to improve recoveries of important flavonoids such as kaempferol, quercetin, and their glucoside derivatives [114]. Flavonoid extraction in MWAE is mainly reported as total flavonoid content (TFC), quercetin equivalent (QE), or total quercetin (TQ) (Table 5).

MWAE has been combined with other techniques in order to improve extraction. These synergistic processes have achieved higher yields in the extraction of flavonoids. Some of these processes include enzyme-assisted ultrasonic microwave (EAUMSE) [155], microwave- and ultrasound-assisted extraction (MUAE) [55, 96], and vacuum microwave hydrodiffusion gravity extraction (VMHG) [172].

### 7.2. Infrared

#### 7.2.1. Infrared: General Description of Equipment and Process

**Figure 10:**
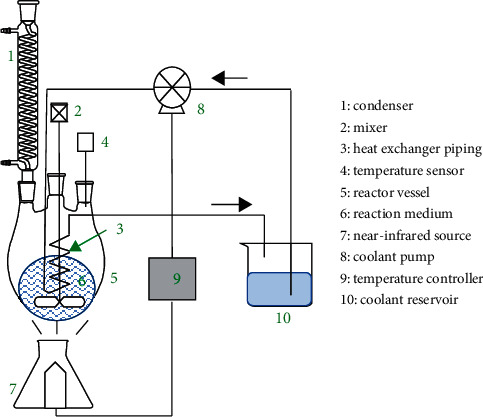
Scheme of a general IRAE equipment.

Equipment for infrared-assisted extraction (IRAE) commonly consists of an IR lamp (Figure 10). This radiation source might be either one lamp with several modes of power or a set of different lamps, where power can be varied from 50 to 1000 W. The plant material is placed inside a vessel like a round-bottom flask with variable volume depending on the solid density. This flask is filled with water, ethanol, or other solvent. Most IRAE instruments are furnished with a condenser for solvent recovery. The flask is placed close to the lamp to expose contents to IR radiation. The IR radiation heats the solvent and plant material, enhancing the extraction of flavonoids. Generally, the extraction is achieved during several minutes.

#### 7.2.2. Main Parameters and Operating Conditions

The main control parameters are type and concentration of solvent, liquid-to-solid ratio, infrared power, and extraction time. The distance of the IR source and operational temperatures are parameters hardly reported in the literature.

*(1) Effects of Solvents*. The comparisons among different solvents such as water, methanol, ethanol, ethyl acetate, and acetone for flavonoid extractions have been reported by Mou et al. [109]. These authors found that methanol and ethanol were the most prominent solvents for extraction of flavonoids. In some studies, it has been concluded that when methanol and ethanol are in higher concentrations, they are less efficient compared with moderate concentrations. This difference has been ascribed to a possible resistance of diffusion due to coagulation of proteins from cell wall when solvents are added in higher concentrations [109, 174].

It has been reported that the use of deep eutectic solvent (DES) improves extraction efficiencies compared with the usage of ordinary solvents like water, ethanol, or methanol. Higher yields of extraction by IRAE with DES are attributed to stronger interactions between hydroxyl or carboxyl groups with polyphenols through hydrogen bonding [175]. Since DES requires the mixing of several compounds which might result in higher costs, an economic comparison between ordinary solvents and DES is needed.

*(2) Effects of Liquid-to-Solid Ratio and Particle Size Influence*. There is also an optimal point in the liquid-to solid ratio, and generally, the extraction enhances when this ratio increases. Overpassing this optimal and the presence of high amounts of impurities in the solvent, such as polysacharides, diminishes the solubility of some polyphenols. For instance, ratios greater than 20 mL/g in the extraction of rutin from *Flos Sophorae* decreased due to impurities inhibiting rutin dissolution [109, 174]. In a recent study, it was reported an increase in extraction yield in 50% for similar liquid-to-solid ratio, and by increasing this ratio, the amount of extraction was constant [176].

The optimum point in liquid-to-solid ratio must be carefully determined in order to discard or to properly consider the diffusion from particle which can also be limiting the extraction. One form to remediate this inconvenience is decreasing the particle size. This can improve the diffusion from solid to solvent as was found for olive leaves when diminishing the particle size from 2 to 0.3 mm which enhanced the extraction yield due to higher contact surface with solvent [176].

As in other methods, such inconveniences can also be remedied by the modification of parameters such as temperature, power, intensity, and time of exposition. The power from IR radiation is therefore related to possible improvements in yield by its interaction with plant matrixes.

*(3) Infrared Interaction with Plant Material and Power Effects*. The knowledge of interaction mechanisms between IR radiation and solids is not completely well understood. Notwithstanding, it is known that energies in near infrared (NIR) ranging from 13000 to 3300 cm^−1^ have been ascribed to possible photon accepting of the *Cytochrome c oxidase* in the cell activities and also ageing human skin [177, 178]; mid-infrared (MIR) ranging 3300–200 cm^−1^ is known for the detection of molecular vibrations in catalytic processes [179] and far infrared (FIR) which ranges from 200 to 10 cm^−1^ is detected by lattice vibrations for instance from metal-oxide compounds [179]. Although reports about photon absorption results in increases in membrane mitochondrial potential when cell is exposed to NIR, far infrared has possible similar effects in mitochondrial membranes besides vibration of cell water. Energies corresponding to FIR frequencies have been used for extraction of flavonoids [180]. Studies of IRAE reported that radiation leads to cell disruption owing to heat effects [180, 181].

The benefits of IR in the extraction of flavonoids have been proved. Duan et al. used far infrared assisted extraction (also known as FIASE) to extract rutin, gentisic acid, and quercetin from *L. barbarum* Linn [180] and concluded that FIASE extracted higher contents of these compounds compared with conventional hot solvent extraction. Moreover, the extraction with FIASE required less than ten minutes compared with hours needed for extraction with conventional hot solvent extraction. Similar results were found by Zhou et al. for the adenosine extraction from *Radix isatidis* [182]; IRAE had a better performance than conventional reflux and maceration. Gan et al. observed a linear increase in the extract content when infrared irradiation time was augmented [183]. They also found that conventional hot solvent extraction required higher extraction times compared with FIASE. This effect was attributed to the heat provoked by the friction from vibration of molecules such as rutin and quercetin, among others, inside the cell and to an interaction between the solids and the solvent which was enhanced by IR radiation. Nevertheless, since lattice vibrations are ascribed in the FIR region, vibration of molecules might not be occurring.

The use of higher voltages does not necessary result in an improvement of extraction. The optimum voltage must be supplied to avoid the boiling of solvent when high power is set [174, 183]. Higher power input leads to an increase in temperature, the oxidation of flavonoids, and consequently lower yields [174].

Adequate control of power and temperature is necessary in order to identify the power effects in the solid. Recently, IR equipment was developed and patented by the Saint-Joseph University of Beirut [184, 185]. In one of the experiments carried out with this equipment, the extraction of polyphenols from orange peels was tested at 60 W of power and 50°C. The time of exposition to IR can be a determinant to increase the extraction of polyphenols but prolonged time might be a detriment to antioxidant properties due to degradation [185]. It is important to note that in this and other recent studies, the exposition to IR is also related with mass. If the amount of mass exposed to IR is excessive, the energy cannot match the solids and interaction of IR radiation with mass is ineffective to some extent [176, 185, 186].

#### 7.2.3. Perspectives of Infrared-Assisted Extraction

Cai et al. compared IRAE with MWAE, UE, and conventional heating extraction processes [181]. Higher extraction and lower processing times were observed when infrared heating was applied. They concluded that conventional heating time was higher due to the heat lost in the air and flask. The main advantage of infrared is that it heats directly the solvent and is suitable for the extraction of important compounds such as catechins and procyanidins. Some studies have shown that the infrared technology improved the extraction compared with MWAE and UE [162, 181].

## 8. Electrical Force Methods

### 8.1. Pulsed Electric Field

#### 8.1.1. Pulsed Electric Field: General Description of Equipment and Process

**Figure 11:**
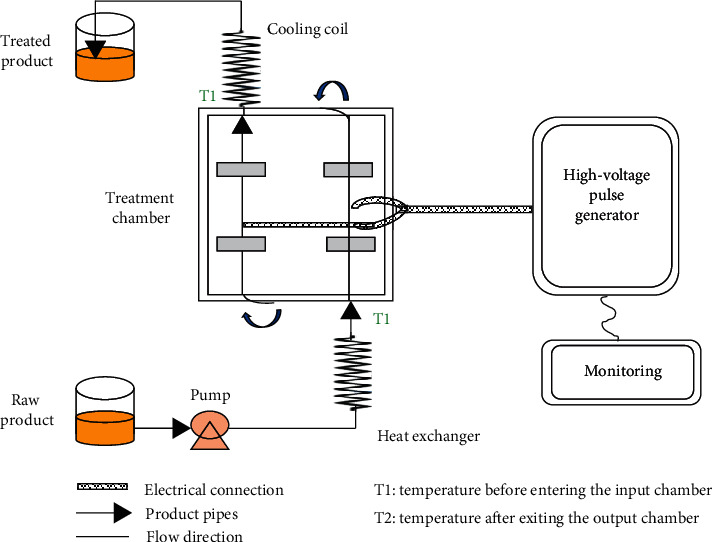
General scheme of a PEF equipment process.

Pulsed electric field (PEF) extraction is a technique based on the exposure of vegetable matrix to an electrical potential. PEF consists in the treatment of the vegetable matrix for the extraction of phytochemicals. Electric pulses are generated by a transformer, increasing voltages from 140 or 220 V to 1000 V or even greater than 25000 V. A capacitor transforms this high voltage in a narrow pulse, and this is achieved in a closed chamber with metallic electrodes. There is no singular weight, shape, or particle size of solids. The chamber size for PEF treatments depends on the solid volume. Thus, PEF has been successfully used to treat whole fruits (or peels) like orange or grapes or slices and cut pieces differing in particle size [187–189]. The electrode plate perimeter might vary from 10 to 100 cm, and the distance between electrodes can be from 1 cm to 20 cm, although the chamber size mostly depends on the volume to treat of the vegetable matrix.  Figure 11 presents a general scheme of a PEF equipment.

#### 8.1.2. Main Parameters and Operation Conditions

*(1) Effects in Plant Material of Energy and Electric Field Strength*. The energy per unit mass (specific energy) is the principal parameter to control in PEF. Energy is a function of electrical potential, electric current, and effective time of pulses. Time of pulses might vary from few microseconds to several hundreds of seconds.

Extraction of flavonoids can be enhanced by PEF at laboratory level [101, 187, 190–193] and also at industrial scale [194]. The enhancement of this method points to the disruption of the cell membrane of the plant matrix. Zimmermann et al. described the rupture of bovine cells by the interaction of electric charges inside the cell, with electric field [195]. In this way, cells are prone to be disrupted, enhancing the formation of internal pores. Pore sizes have been correlated with the intensities of the electric fields. Thus, lower intensities of electric fields produce small pores whilst higher intensities lead to irreversible cell breakdown and larger pores [196]. This process is also known as electroporation which is defined as the permeability of cell membrane by the action of an external electric field. It has been concluded that higher tissue damage increases both permeability and electroporation. The cell damage depends on the electric field strength (electric field), capacitance of discharge capacitor, and pulse number [197]. Contrary to lower electric fields (≈13 kV/cm), high-voltage electric discharges such as 40 kV/cm produce a greater fragmentation of solids like mango peels due to a process of cavitation and bubble explosion, achieving higher degree of extraction [192]. However, extraction yields are related to optimum values of electric field strength [198].

*(2) Cell Damage Measurements and Permeability*. The cell damage can be characterized by means of electrical conductivity disintegration (*Z*_c_), acoustic disintegration (*Z*_a_), and cutting disintegration (*Z*_f_) indexes.

These indexes are strongly related to electric field strength, and for the same number of pulses, increasing the electric field strength increases the index of disintegration [198]. Peiró et al. exposed lemon residues to PEF as pretreatment and found that less than 5 kV/cm did not show significant cell damage (*Z*_c_ < 0.2) whilst values of 7 kV/cm increased the index to 0.5. Higher rates of extraction were obtained with the lemon residues treated with 7 kV/cm, and clearly, by increasing this index, the rate of extraction should also increase. Solids with similar disintegration indexes differ in their rates of extraction due to other causes. For instance, significant differences in kinetics of extraction for apple fruits with similar acoustic disintegration indexes (*Z*_a_ > 0.8) were related to apple fruit shape and size. The same authors concluded that for apple slices, PEF was more uniform whilst in the whole fruit, PEF only affected the external surface [199]. Moreover, small particle sizes, for instance, from sawdust, generally lead to a higher extraction yield.

Bouras et al. carried out PEF with solvent in the equipment. Solvent with basic pH achieved higher extraction yields compared with acid pH [193]. This was attributed to a weak acid character of polyphenols contained in sawdust. Notwithstanding, acidic medium was found to be efficient for total phenolic compound extraction by conventional methods [191, 192].

Permeability can be assessed by centrifugation in dry condition. Comparison and evaluation of the degree of permeability and enhancement of extraction are usually achieved between non-PEF-treated (or control sample) and PEF-treated counterparts [88, 200]. Moreover, increasing PEF intensity caused higher permeability and consequently the enhancement of extraction of intracellular compounds [201]. Other reports have related the observed improvements to the dissolution of phospholipids with the organic solvent [188].

*(3) PEF Effects on Properties of Bioactive Compounds*. PEF can also modify the properties and quality of bioactive compounds; for instance, it has been reported that this technology increases the antioxidant levels of fruits and grains [190, 202–205]. As a consequence of PEF exposition, the vegetable matrix maintains the secondary metabolism aimed towards the autoprotection by increasing the synthesis of phenolic compounds and other secondary metabolites and also by the chemical stabilization of flavonoids [196, 206]. El Darra et al. found an increase in flavonoid production due to an autoprevention mechanism caused by chemical agents such as oxygen [187]. On the other hand, PEF treatment is associated with the higher enzymatic oxidation of polyphenolic compounds and the more efficient extraction of flavonoids [200]. However, the application of PEF can also be detrimental due to changes in density and color of fruit products [207]. Delsart et al. working with wine concluded that PEF caused enzyme degradation which increased density [208]. The effects of PEF in terms of modification of enzymatic activities depend on the initial concentration of compounds as reported by López-Giral et al [190]. The PEF intensities also influenced secondary metabolism activities because of the reported degradation of phenolic compounds [201, 202]. More studies are needed in order to understand the mechanism of interaction of PEF with plant matrixes and to evaluate the possible degradation due to the action of high temperature and the chemical medium.

#### 8.1.3. Perspectives of Pulsed Electric Field

The main uses of PEF in the food industry are for juice production, enhancement of oil extraction from cottonseed, and enhancement of the shelf-life by microbial inactivation in beverages such as milk, beer, and fruit juice. The PEF technology has also been used to enhance the extraction yield of sucrose from sugar beets. Despite these myriad commercial applications, PEF extraction has been subjected to exhaustive scientific research in order to better understand its effects on different vegetables matrixes and to optimize processing conditions especially in terms of autoprotective mechanisms. Thus, profound and more intensive investigations of PEF in the extraction of flavonoids are needed.

### 8.2. High-Voltage Electrical Discharges

#### 8.2.1. General Description of High-Voltage Electrical Discharge Equipment

The principal components of the high-voltage electrical discharge (HVED) equipment are (a) a high-voltage generator (20–40 kV), (b) two electrodes, (c) oscilloscope, and (d) a discharge chamber. Inside the chamber, the solvent-solid mixture is placed and covered by lid; the electrode is in direct contact with this mixture. This ensures the discharge generated to reach the solvent and the solid. The discharges might be from 20 to 40 kV, and the current is adjusted as required. The oscilloscope measures the generated pulses. The principle of this equipment is similar to PEF, with the difference that electrical discharge is made in a small point. For this, a needle electrode is used from which the discharge is made in a plate grounded electrode. The simplest mode of operation is the batch. However, there are HVED instruments which were designed to operate in continuous and recirculation mode. In reference [209], very good schemes of these three modes are presented.

#### 8.2.2. Main Parameters and Operating Conditions

*(1) Specific Energy*. It is found in several studies that the increase in specific energy leads to an increase in the amount of substance withdrawn, and normally, it is found an optimal voltage peak in which the greatest amount is extracted [210, 211]. Subsequently, there is a decrease in the amount of substance withdrawn when specific energy is increased beyond the peak of voltage. Although this decrease in the amount extracted with increasing energy has not been completely explained, it is likely to occur due to the limitation of the mass to be treated. That is, the energy cannot match the mass adequately. However, the effect of the electric shock action is also not fully elucidated. On the one hand, the discharge of electrons from the electrode can first have effects on the fluid (solvent liquid and gas). Although, ionized gas can be formed from the electrons' interactions with gas, the effects that seem to be more influential come from the same fluid, and in this case, the hydrostatic pressure plays an important role [212]. When the hydrostatic pressure is low or the vapor pressure of the fluid is high, pockets of vapor and gas are formed. This compressible fluid can generate shockwaves, which could be more responsible for damage to the solid material. Furthermore, the gas which can mainly be air, when becomes ionized and forms radicals, can oxidize the extracts. A complete study about how the energy is affecting the solids is needed in order to understand its implications on the selectivity, the amount of the extracts, and antioxidant properties.

*(2) Electrode Distance*. The distance from the electrode to the discharge plate normally influences the extraction performance. In general, very large distances weaken the intensity of the electric field. In other words, the energy seems to have no effects on the plant material, so it cannot be altered. Also, very short distances reduce the time in which the electric arc of the discharge is formed, so the effects of energy on the plant matrix are also very weak. For this reason, there should normally be an optimal distance, although this will depend mainly on the type of solvent. Most of the studies reported a distance of the electrodes to be 5 mm long [101].

*(3) Liquid-to-Solid Ratio*. Most HVED extraction studies focus on having the minimum liquid-to-solid ratio; this is the one where the solid mass can be completely immersed in the liquid. There are also works in which the optimal value in the liquid-to-solid ratio has been studied. Normally, the extraction level of the substances of interest increases when this ratio (L/S) increases [53]. This is normal since the molecular diffusion should be greater when more solvents are available. Boussetta et al. found that the L/S ratio of 5 was optimal to extract polyphenols from grape residues from vineyards [101]. After a maximum value of L/S ratio, the extraction reaches a stable level, which seems to have reached an equilibrium. For this reason, it would be recommended to carry out these studies of optimal L/S ratio, especially when considering scaling the operation to an industrial level. However, the diffusion of the substances of interest from the plant material to the solvent can also be a function of temperature, and therefore it might be helpful to study this parameter as well by thermodynamic equilibrium studies.

*(4) Type of Solvent and pH of Dissolution*. Usually, the solvents used in the HVED technique are water and ethanol or mixtures of these. Those that have been more successful when it comes to the level of flavonoids extracted are mixtures whose concentration of ethanol is approximately 30% by volume [53]. Although ethanol-water mixtures are mostly used for their effectiveness, DES and other solvents such as lactic acid and glycerol have also been tested. The extraction of flavonoids has also been favored in these mixtures thanks to the fact that the DES contains components such as chlorides-choline and lactic acid that can be linked by hydrogen bonds with the polyphenols; in addition, the effectiveness in the solubility in mixtures of glycerol-water can be explained due to the electrical permittivity of water which is modified by glycerol favoring the solubility of polyphenols [213]. In this sense, glycerol is a good prospect to be used as a solvent owing to its nontoxicity and low cost with relevant results in the extraction of flavonoids in other methods such as homogenizer-assisted extraction [214].

The extractions that are made by varying the content of ethanol in the solvent lead to pH changes in the solution. For this, in some works, hydrochloric acid and sodium hydroxide have been used to maintain the pH at a fixed value. Acid pH tends to be the means that favor the extraction of the substances of interest [101, 191]. Acid solutions are reported to promote the solubility of phenolic compounds and produce the degradation of cellular material. Furthermore, the oxidizing effect of the radicals generated during the electric shock could be minimized by the low pH [215].

#### 8.2.3. Perspectives of HVED

This technique has been used in combination with enzymatic hydrolysis. In a study by El Kantar et al., stimulation of enzymes was achieved through the action of electric shocks by the HVED equipment to extract phenolic compounds from orange peels. The results were very encouraging since a beneficial effect was found when combining HVED and enzymatic hydrolysis in acidic medium. Specifically, the extraction of polyphenols and reduction of sugars were favored, which also come from this type of vegetable matrixes. This could potentially replace other pretreatment methods for the extraction of reducing sugars such as stem explosion, acid pretreatment, and popping pretreatment whose purpose is the enzymatic production of biofuels [216].

The main advantage of HVED is the operability in continuous mode which is very important from industrial and economic point of view. The converged electric field HVED equipment operates in continuous mode and one of the inconveniences is that the discharge is made in a hole of 1 mm diameter drilled in the middle of an insulating plate between the electrodes. The problem of this is the small ducts and discharge hole where high amounts of solids might clog the system. The annular space type is other continuous mode HVED equipment which consists of two concentric electrodes with annular space opened with enough space to avoid obstruction from solids. However, sometimes, efficiencies are lower than those in converged electric field type. The equipment with recirculating system has the capacity to improve yields because solids are exposed to electrical discharges several times. Consequently, the HVED technique is an interesting method whose principal advantage is that it can be used as pretreatment or as a direct method of extraction with very high yields.

## 9. Enzymatic Reaction Systems

### 9.1. Enzyme-Assisted Extraction

#### 9.1.1. General Description of the Method

Enzyme-assisted extraction (EAE) is a method based on the biocatalytic activity of diverse enzymes. The basic principle of EAE is the breaking of the cell wall of the plant by the action of one or various enzymes. The enzymes catalyze the hydrolysis of cell walls, releasing intracellular components [150].

The enzymes have an active site, which is an area where the substrate is linked to be catalyzed. The substrate is a molecule on which the enzyme acts. In this case, the substrates are found in the plant cell walls, which are composed of a series of polysaccharide complexes such as cellulose, hemicellulose, pectin, lignin, and some proteins. All these components confer to the cells' stability and resistance to the extraction of the bioactive compounds from the intracellular components. Generally, the substrate binds to the enzyme by coupling to an active site, causing a conformational change in its structure and a deep interaction with the substrate (cell wall). This leads to the breaking of the bonds of the cell wall and the release of its bioactive components. Therefore, the EAE process uses a wide range of carbohydrate hydrolyzing enzymes and may be either a single or a preparation of several enzymes. Enzymes such as pectinases, cellulases, and hemicelluloses hydrolyze the components of the cell wall, increasing the permeability and the extraction yield of specific compounds such as oils, polyphenols, polysaccharides, and pigments, among other medicinal compounds [150, 217, 218].

Enzymatic processes have proven to be efficient for the extraction of polyphenols, especially anthocyanidins and flavonoids from their respective glycosides [219]. Some phytochemicals are dispersed in the cell cytoplasm of the plant, and some others are retained in the polysaccharide-lignin network by hydrogen bonds and are sometimes inaccessible to the solvents used in conventional methods.

*(1) Enzyme Types*. Enzymes such as xylanase, protease, amylase, papain, and hemicellulose have been employed in different EAE processes. There are several types of enzymes, which are classified as hydrolyzing, oxidation-reduction, group transfer, isomerizing, and carboxylation enzymes. Each of these enzymes acts on a specific substrate, depending on its catalytic reaction characteristics [219].

Generally, the intracellular biomolecules present in plant materials are found as insoluble substances or soluble conjugated forms (glycosides). For example, phenolic compounds are linked to the cell walls by hydrophobic interactions and hydrogen bonds. Some phenolic acids form ether bonds with the lignin through the hydroxyl groups present in the phenolic aromatic ring, and other acids form ester bonds with the carbohydrates and proteins of the cell wall by carboxylic groups. Some polyphenols are present as glycosides; these are polyphenols covalently linked to glucose segments. In the case of flavonoids, these are bound by covalent bonds with glucose sections through the OH (O-glucose) groups or through carbon-carbon (C-glucose) bonds. The enzyme *β*-glucosidase has the ability to break glycoside bonds *β*-1,4. In addition to *β*-glucosidase, pectinases also hydrolyze the cellulose, hemicellulose, and pectin solubilizing cell wall components and accelerate the release of intracellular biomolecules [150].

#### 9.1.2. Main Parameters and Operating Conditions

In the EAE method, there are parameters that must be established to optimize extraction yield, for example, (1) particle size and moisture content of the plant material, (2) type and concentration of the enzyme, (3) extraction time, (4) the pH of the system, and (5) extraction temperature [150, 220].

*(1) Particle Size and Moisture Content of Raw Material*. The preliminary treatment of the plant plays an important role in EAE. Preliminary treatments include reduction and uniformization of the particle size and removal of moisture. These treatments affect the extraction yield and mass transfer of bioactive compounds. Generally, a high content of antioxidant properties in the extracts is associated with small particle diameters. However, if the particle size is very small, there will be greater availability of bioactive compounds, which can be degraded during cell wall rupture [18]. In general, dehydration removes the water bound to the plant, increasing the porosity of the cellular material. This improves the enzyme-substrate contact and facilitates the rate of diffusion which increases the recovery of phytochemicals in the final extract.

*(2) Enzyme Type and Concentration*. The selection of the enzyme and its concentration depends on the composition and nature of the specific plant cell wall. A mixture of enzymes ensures a complete fragmentation of the cell wall. The enzymatic specificity on a specific substrate is determined by the structure and the active site of the enzyme. When the substrate concentration is high, the enzyme addition can accelerate the reaction rate, until the substrate concentration becomes limiting. Santana and Macedo used pectinase (P), cellulase (C), and a P + C mixture for the catechin recovery of waste guarana seeds at 40 and 50°C. They found a higher recovery % with the enzyme pectinase, at 40°C, concluding that the mixing of the two enzymes does not contribute to a higher catechin extraction yield, at both temperatures [18]. However, Roggia et al. used the mixture of tanasse (T) and *β*-glucosidase (B) to extract flavonoids from citrus juice by-products, achieving an increment in the yield. They also observed that the recovery of naringenin, hesperetin, and diosmetin was higher when they used the enzymatic mixture. These results indicated a synergistic effect between T and B [221].

*(3) Extraction Time*. The solubility of the cell wall components increases when the exposure time between the substrate enzyme and the solvent is prolonged. However, on a large scale, extended times would result in low quality extracts and energy inefficiency.

*(4) Optimal pH Conditions*. In each enzymatic process, the optimum pH must be selected for the maximum activity. Most extractions are carried out under acidic pH conditions (Table 6). This is because the acid medium destabilizes the hydrogen bonds that link bioactive compounds and the plant cell wall. This favors the rupture of the cell wall.

*(5) Extraction Temperature*. Enzymatic reactions are usually performed at low temperatures, between 15 and 45°C (Table 6). At temperatures over 60°C, most enzymes are irreversibly altered by the action of heat. It is important to select the appropriate temperature for the extraction since a very low temperature will not accelerate the enzymatic reaction, which leads to a lower extraction efficiency.

#### 9.1.3. Synergy Effects between EAE and Other Methods

There are reports, where a combination of several extraction methods was made with EAE. Xu et al. combined EAE with ultrasound and microwave methods for the recovery of flavonoids from chestnut peels. They found that the combination of these three techniques improved the flavonoid extraction yield, compared to the individual technique. In addition, they evaluated the effect of pH and observed that the maximum recovery of TFC (total flavonoid content) was 1.08% (w/w) with the enzyme pectinase, at pH 5 [155]. Tchabo et al. combined ultrasound extraction with EAE for the extraction of flavonoids from mulberry. They obtained a yield of 378.02 mg/100 mL of TFC using a frequency of 34 kHz, at 20°C for 12 min, with the enzyme Pectinex UF® [230]. Similarly, Balasubramaniam et al. performed a pretreatment of the plant material (finger millet variety) with xylanase, before performing the ultrasound extraction. They observed a yield of polyphenols 20% higher than that shown only by ultrasound. They also found that the extracts of the plant material pretreated with cellulose contained less polyphenols [231]. Park et al. performed the enzymatic extraction of flavonoids from rice hull in combination with HHPE. In the extracts, a TFC of 245.6 ± 8.12 mg/kg was obtained, with the enzyme Pectinex ®, at 40°C, for 3 h, at pH of 5 [232]. More recently, Cascaes Teles et al. combined EAE with high hydrostatic pressure for the extraction of phenolic compounds from grape pomace. They found that the use of HHP increased the activity of the enzymes used in the extraction up to 16 times [233]. Van Hung et al. recovered naringenin and hesperidin from pomelo peels using the combined enzyme and ultrasound-assisted extraction. They concluded that the combination of both methods was effective for bioactive compound extraction with the highest antioxidant and antimicrobial activities [234].

#### 9.1.4. Perspectives of Enzyme-Assisted Extraction Method

An important advantage of EAE is that the enzyme acts on a specific substrate. In addition, this methodology accelerates the release of phytochemicals from resistant and compact materials, such as bark and roots [150, 218, 219].

EAE has not gained acceptance at industrial scale due to the high cost of enzymes. Moreover, slight modifications in the medium such as oxygen concentration, pH, nutrients, and temperature lead to a possible alteration in specificity or complete deactivation of enzyme.

## 10. Conclusions

### 10.1. General Conclusions

In this work, the main parameters and operating conditions of unconventional methods for the extraction of flavonoids were analyzed. Such techniques included UE, PLE, MAE, HHPE, EAE, SFE, MWAE, IR, PEF, NPCE, and HVED. These techniques represent the attention of mankind to improve the quality of life and the good preservation of the environment. They have also represented the ability of researchers to create innovative processes to improve the extraction of flavonoids from plant material. Most of these techniques exhibit adequate efficiency of extraction and preserve or improve the antioxidant properties.

Despite the large number of parameters studied, such as specific energy, temperature, and solvent type, and the possibility to obtain the optimum conditions of extraction, solvent-flavonoid interactions are not well understood. For a better understanding, it would be important to conduct thermodynamic studies because only a few were found in the literature. Thermodynamic studies might help to complete the knowledge of equilibrium and the limits of extraction at different temperatures. Temperature turned out to be the most important parameter due to two principal effects: (a) the decrease in viscosity of solvent normally favors the solubility of solutes and (b) the antioxidant properties of flavonoids can be lost at temperatures above 50°C. Notwithstanding the advantage of the analyzed unconventional methods is the possibility of increasing temperature and in most of the works reviewed, rising the temperature generally improved the yield. In kinetic studies, the relevance of temperature was embodied as in UE and MWAE methods. Other kinetic studies showed the importance of other parameters such as the strength of electric field. In PEF, this parameter is important to increase the rate of extraction. However, more profound kinetic studies need to be done which will help to understand the mechanism of solvent-solute interaction and improve the yield and selectivity.

Like temperature, the energy input is also an important parameter. Most of the techniques analyzed converge in the nonuniform energy transmission to the medium. Thus, certain amount of energy is lost when interacting with the surroundings. Examples of these techniques are UE, IR, PEF, and HVED.

### 10.2. Specific Conclusions for Each Unconventional Method

In the UE method, in most of the works, there are no reports of important parameters such as power and intensity, and thus it is hard to understand how they can influence cavitation with the damage of cell wall.

Pressure is the most important parameter in PLE owing to its effects in the contact between solvent and solute, and in some cases, this interaction is not reached in atmospheric processes.

The MAE and HHPE methods operate at environment temperature which was an advantage to preserve antioxidant properties of flavonoids. In MAE, acidic medium is important to recovering flavonoids. However, the MAE method is not completely developed, and there were no kinetic studies found to understand the extraction mechanism. In HHPE, the pressure allows the rupture of cell and therefore is an important parameter. The HHPE technique needs more studies for separation and purification of extracts.

The action of temperature in the SFE technique turned out to be more important than pressure. So, temperature has a strong impact on solvent viscosity, and therefore the solubility of solutes might change constantly. This is remedied by the addition of modifiers to the principal solvent for this method, CO_2_. However, in SFE, the parameters are intimately linked, which is difficult for the operability with all these variables.

In the NPCE method, the temperature increase has negative effects on flavonoid extraction. This is due to the fact that the solvent might volatilize so fast and the solid-solvent contact and therefore the solubility might decrease. Negative pressure is an important parameter because it promotes more collisions between solids which improve the yield.

In MWAE, the effect of heating is related to the improvement of extraction. In this technique, a lack of information of parameters which are not reported was also found. Power of MW was the principal parameter to affect the yield, and the understanding of MW-solid remains ambiguous. In MWAE, the solvent might be important in terms of dielectric properties, which influences the extraction. Specifically, carbonyl species were found to be extracted with relative ease by the action of MW. However, MWAE has also been achieved without a solvent, and a clear comparison of studies with solvent and without solvent was not found in the literature.

Similar conclusions as MWAE can be drawn for IRAE. The IRAE is a method in which extraction of flavonoids has been ascribed principally to its effects of heating, and the most important parameter was the power of source. However, some studies reported that inside the plant material, interactions among IR waves and flavonoids may be occurring. Therefore, profound studies are necessary and, in this sense, although it is a hard task, a better control of irradiated wave could be helpful to create a narrow effect in solids. The IRAE also was found to improve the extraction of flavonoids when it is used as a pretreatment method.

The electric field strength was the most important parameter in PEF and HVED due to its effects in cell damage. It also has effects in fluids which might be multiplying the damage effect in solids by the formation of cavitation bubbles and the shockwaves when they collapse. Other important parameter in PEF was the disintegration index which is related to permeability owing to electroporation. However, measurements of the pore size and its correlation with electric field strength were not found in the literature.

Finally, one of the major advantages of the EAE method is the rapidness of extraction with high selectivity. However, inconvenience of EAE is the extreme attention to parameters such as pH and temperature which might inactivate the enzymes.

## Figures and Tables

**Table 1:** Representation of the most common extraction conditions and equipment used in the different conventional methods.

Equipment	Most used solvents/temperature and time range	Reference
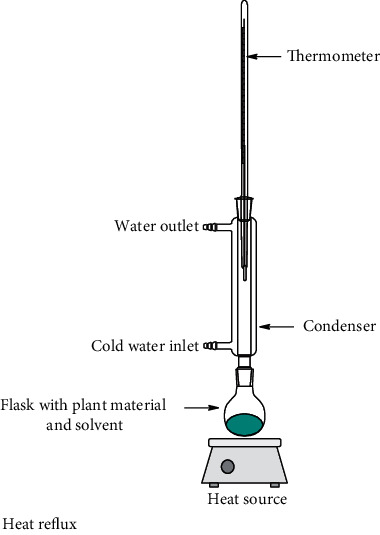	Water Ethanol (EtOH) Methanol (MeOH) EtOH-Water mixture (50–90%) MeOH-water mixture (60–70%) 60–95°C 15 min–12 h	[51, 55–58]
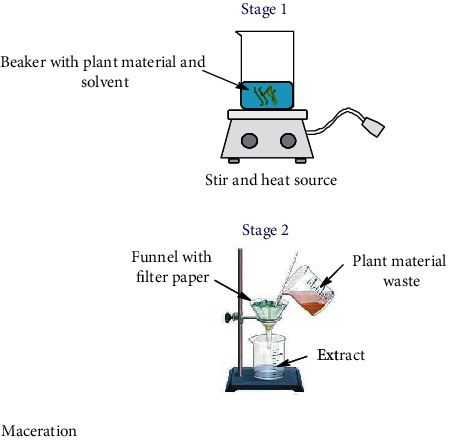	EtOH MeOH EtOH-water mixture (30–90%) MeOH- water mixture (60–90%) 20–60°C 2–72 h	[53, 57, 59–65]
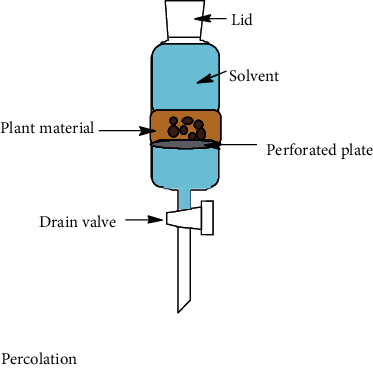	Water EtOH MeOH EtOH-water mixture (50–95%) MeOH-water mixture (80%) 10–25°C 24–72 h	[66–70]
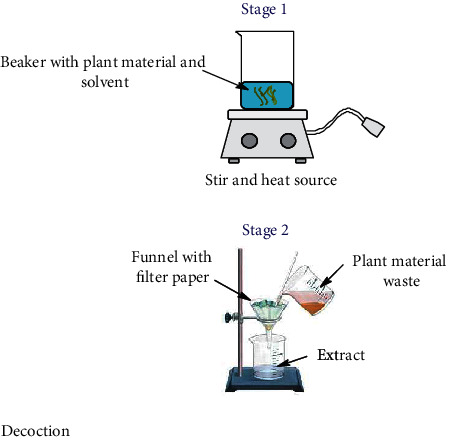	Water 100°C 5–30 min ^*∗*^Used for hard plant material such as roots, barks, stems, and seeds	[71–75]
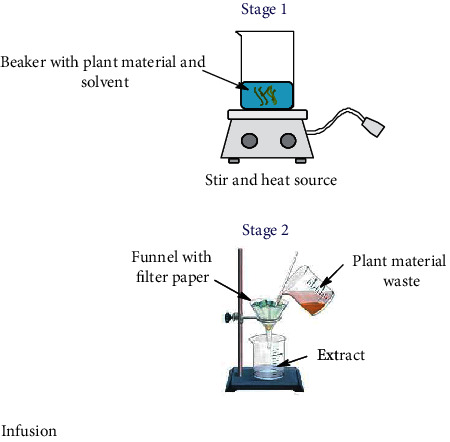	Water 100°C 5–30 min ^*∗*^Used for soft plant material such as flowers and leaves	[74–77]
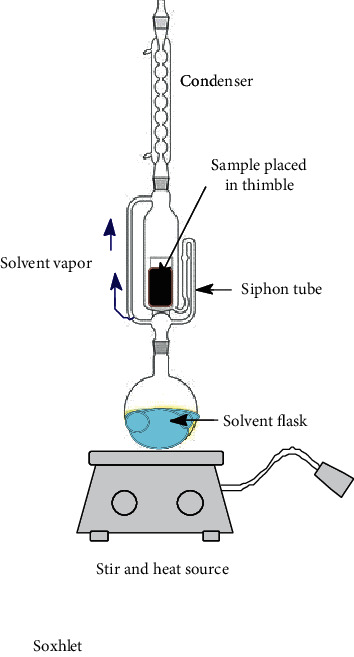	Water EtOH MeOH EtOH-water mixture (50–96%) MeOH-water mixture (60–96.6%) Petroleum ether Acetonitrile Acetone Ethyl acetate Dichloromethane Hexane Boiling point of solvent 2–48 h	[59, 62, 64, 69, 73, 78–83]

**Table 2:** Conditions of the extraction of flavonoids by ultrasound.

Source	Experimental conditions	Flavonoid	Yield	Reference
*T. inflorescentia*	Solv: 80% MeOH Temp: 60°C Time: 20 min Frequency: — Power: — Liquid-solid ratio: 25 mL/g	Rutin Isoquercetrin Astragalin Tiliroside	565.49 ± 12.69 *μ*g/g 118.47 ± 5.64 *μ*g/g 63.55 ± 1.10 *μ*g/g 910.28 ± 26.82 *μ*g/g	[92]
*Calligonum comosum*	Solv: MeOH, EtOH Temp: 30°C Time: 30 min Frequency: — Power: 720 W Liquid-solid ratio: 10 mL/g	TFC	0.51 ± 0.14 mg/g (MeOH) 32.97 ± 0.78 mg/g (EtOH)	[93]
Mandarin peel	Solv: 80% Ac Temp: 48°C Time: 40 min Frequency: — Power: 56.71 W Liquid-solid ratio: 20 mL/g	Hesperidin	6435.53 mg/100 g	[63]
*Arbutus unedo* L. fruits	Solv: 58.8% EtOH Temp: — Time: 28.6 min Frequency: — Power: 246.7 W Liquid-solid ratio: 20 mL/g	TFC	29.49 ± 0.72 mg/g	[65]
*Selaginella helvetica*	Solv: 95% EtOH Temp: 47°C Time: 40 min Frequency: — Power: 250 W Liquid-solid ratio: 12.7 mL/g	Biflavonoids	Approx. 12.1 mg/g	[69]
*Camellia oleifera* seeds	Solv: 60% EtOH Temp: 30°C Time: 30 min Frequency: — Power: — Liquid-solid ratio: 50 mL/g	TFC	15.91 ± 0.86 mg/g	[80]
*Nectandra grandiflora*	Solv: 96% EtOH Temp: 50°C Time: 30 min Frequency: — Power: 750 W Liquid-solid ratio: 20 mL/g	TFC	114.50 ± 0.71 mg/g	[81]
*Hypericum formosanum* tissues	Solv: 73.5% EtOH Temp: 62.5°C Time: 38.3 min Frequency: 40 kHz Power: — Liquid-solid ratio: 100 mL/g	TFC	101.7 ± 1.7 mg/g	[94]
*Amaranthus paniculatus* leaves (a), defatted seeds (b), and non-defatted seeds (c)	Solv: 90 : 10 MeOH: EtOH Temp: 25°C Time: 50 min Frequency: — Power: — Liquid-solid ratio: 15 mL/g	Rutin	3.62 ± 0.06 g/kg (a) 0.19 ± 0.001 g/kg (b) 0.34 ± 0.002 g/kg (c)	[95]
*Oryza sativa* cv. Poireton	Solv: 67.34% EtOH Temp: 49.46°C Time: 10.02 min Frequency:— Power: — Liquid-solid ratio: 40.79 mL/g	TFC	3.01 mg/100 g	[96]
*Pistacia vera* L. hulls	Solv: MeOH/water/FA (80 : 19 : 1) Temp:— Time:— Frequency:— Power:— Liquid-solid ratio: 60 mL/g	Total flavonols	3.38 ± 0.18 g/kg	[97]

Solv: solvent, Temp: temperature, TFC: total flavonoid content, EtOH: ethanol, MeOH: methanol, Ac: acetone, and FA: formic acid.

**Table 3:** PLE of flavonoids (general conditions of extraction).

Source	Experimental conditions	Flavonoid	Yield	Reference
*Vitis vinifera* L. cv. Cabernet Sauvignon seeds (a) and *Vitis vinifera* L. cv. Tempranillo seeds (b), pomace, (c) and stems (d)	Solv: water Temp: 120°C Time: 10 min Pressure: 1500 psi Number of cycles: 2 Flow rate: —	Flavonols	2.66 ± 0.11 mg/g (a) 1.62 ± 0.03 mg/g (b) 15.74 ± 0.66 mg/g (c) 0.34 ± 0.24 mg/g (d)	[105]
*Tilia cordata* inflorescence	Solv: 80% MeOH Temp: 80 and 120°C Time: 30 min Pressure: 60 bar Number of cycles: 3 Flow rate: —	Rutin Isoquercetrin Astragalin Quercitrin Tiliroside	582.20 *μ*g/g (80°C) 621.15 *μ*g/g (120°C) 81.56 *μ*g/g 85.18 *μ*g/g 60.80 *μ*g/g 69.05 *μ*g/g 190.73 *μ*g/g 202.80 *μ*g/g 903.37 *μ*g/g 1038.34 *μ*g/g	[92]
*Camellia oleifera* seeds	Solv: water Temp: 140°C Time: 10 min Pressure: 600 psi Number of cycles: — Flow rate: —	TFC	18.80 ± 0.38 mg/g	[80]
*Momordica charantia* fruit	Solv: 80% EtOH Temp: 160°C Time: 10 min Pressure: 10 MPa Number of cycles: 3 Flow rate: —	TFC	1.50 ± 0.10 g/100 g	[56]
*Passiflora edulis* f. *flavicarpa* leaves	Solv: 64% EtOH Temp: 80°C Time: 10 min Pressure: 1500 psi Number of cycles: 5 Flow rate: —	Isoorientin Orientin Isovitexin	0.58 ± 0.004 mg/g 0.26 ± 0.004 mg/g 2.22 ± 0.030 mg/g	[111]
Cocoa	Solv: water Temp: 125°C Time: 3 min Pressure: — Number of cycles: — Flow rate: —	Catechin Epicatechin	88.8 ± 5.5 mg/100 g 23.0 ± 2.7 mg/100 g	[112]
Olive leaves	Solv: 50% EtOH Temp: 100°C Time: 15 min Pressure: 10.34 MPa Number of cycles: 1 Flow rate: —	TFC	16.51 mg/g	[113]
*Moringa oleifera* leaves	Solv: 35% EtOH Temp: 128°C Time: 20 min Pressure: 10 MPa Number of cycles: 1 Flow rate: —	Quercetin	0.2 mg/g	[114]
*Salvia officinalis*	Solv: water Temp: 140°C Time: 30 min Pressure: 10.3 MPa Number of cycles: — Flow rate: —	Quercetin 3-gl Luteolin 7-O-*β*-D-gl Apigenin 7-O-*β*-D-gl	1270 ± 89 *μ*g/g 4364 ± 326 *μ*g/g 1698 ± 9 *μ*g/g	[115]

Solv: solvent, Temp: temperature, TFC: total flavonoid content, EtOH: ethanol, MeOH: methanol, Quercetin 3-gl: quercetin 3-glucuronide, Luteolin 7-O-*β*-D-gl: luteolin-7-O-*β*-D-glucuronide, and Apigenin 7-O-*β*-D-gl: apigenin-7-O-*β*-D-glucuronide.

**Table 4:** Experimental conditions for supercritical fluid extraction of flavonoids.

Source	Experimental conditions	Flavonoid	Yield	Reference
*Salvia officinalis*	Solv: CO_2_-water Flow rate: 2 L/min Temp: 60°C Time: 10 min Pressure: 45 MPa	Quercetin 3-glucuronide	1270 ± 89 *μ*g/g	[115]
*Morus alba* (a) and *Morus nigra* (b) leaves	Solv: CO_2_ Flow rate: 0.194 kg/h Temp: 40°C Time: 17 h Pressure: 300 bar	TFC	22.5 ± 0.7 mg/g (a) 43.5 ± 0.1 mg/g (b)	[59]
*Odontonema strictum* leaves	Solv: CO_2_-EtOH (85 : 15) Flow rate: 15 g/min Temp: 65°C Time: 270 min Pressure: 200 bar	TFC	230.43 mg/g	[138]
*Abelmoschus manihot* L. flowers	Solv: CO_2_-90% EtOH Flow rate: 2 L/min Temp: 60°C Time: 10 min Pressure: 20 MPa	TFC	41.58 mg/g	[141]
*Cissus sicyoides* L. leaves and stems	Solv: CO_2_-EtOH (10%) Flow rate: 4.52 g/min Temp: 40°C Time: 3.5 h Pressure: 400 bar	TFC	12.13 ± 0.29 mg/g	[142]
Spina gleditsiae	Solv: CO_2_ Flow rate: — Temp: 48°C Time: — Pressure: 40 MPa	TFC	0.793%	[143]

Solv: solvent, Temp: temperature, TFC: total flavonoid content, EtOH: ethanol, and MeOH: methanol.

**Table 5:** Flavonoid sources and principal parameters and conditions in MWAE.

Plant^a^	Solvent^b^ (concentration)	Conditions during extraction^c^	Results^d^ yield and optimum conditions	Reference
*Arbutus unedo* L. M: 1 g P.S.: —	Ethanol (0–100%)	P: 400 W TOE: 1.6–45 min S.C.: —	Y: 81.23 ± 6.34 mg/g O.C.: 1.6 s, —, 120 ± 10.9°C	[65]
*Nectandra grandiflora* leaf M: 5 g P.S.: —	Ethanol (96%)	P: — TOE: 30 min S.C.: 50°C	Y: 123.83 ± 3.60 mg QE/g of dry weight O.C.: —	[81]
*Oryza sativa* c.v. (black rice) M: 2 g P.S.: 0.841 mm	Ethanol (40–70%)	P: — TOE: 20–60 s S.C.: sample first was exposed to ultrasound-assisted extraction	Y: 3.04 mg of TFC/100 g O.C.: 31.1 s, 1 g/40.79 mL, —, —	[96]
*Eleocharis dulcis* (chestnut peels) M: 2 g P.S.: —	Ethanol	P: 100–300 W TOE: 30–180 s S.C.: sample treated before extraction with enzymatic hydrolysis and 0.1 mol/L of NaH_2_PO_4_ with varying pH, enzyme and NaH_2_PO_4_ concentration, time, and power	Y: 1.48% of TFC (w/w) for EAUMSE O.C.: 60 s, 200 W for EAUMSE	[155]
*Satureja macrostema* M: 3 g P.S.: —	Ethanol (0–100%) Water	P: — TOE: — S.C.: ultrasound process was accoupled (microwave- and ultrasound-assisted extraction)	Y: 123.88 ± 8.62 mg of TFC/g O.C.: —	[55]
*Theobroma cacao* L. (cacao leaves) M: 1–6 g P.S.: 0.1–0.6 mm	Ethanol (85%)	P: 100–800 W TOE: 4–35 min S.C.: — S.C.: 20–80 mL/g of solvent-to-solid	Y: 80–95% O.C.: —	[156]
Tomato M: — P.S.: —	Ethanol (0–100%)	P: 200 W TOE: 0–20 min S.C.: —	Y: 11.7 ± 0.6 mg of TFC/g O.C.: 2 min, 5 g/L, —, 60°C, ethanol (100%)	[157]
*Physalis angulata* M: — P.S.: 10-mesh sieve	Ethanol (0–100%)	P: 10–30 W TOE: 30–50 s	Y: 0.86 mg of rutin/L O.C.: 50 s, 30 mg/L, —, —, 30 W, 50% Y: 2.43 mg of mangiferin/L O.C.: 50 s, 20 mg/L, —, —, 10 W, 100%	[158]
Young barley leaves M: — P.S.: 40-mesh sieve	Water	P: 0.4–1.32 W per gram TOE: 4–20 min S.C.: —	Y: 80.78% as rutin equivalents O.C.: 11 min, 34.02 mL/g, 1.27 W per gram	[159]
*Allium cepa* L. (onion peels) M: 1 g P.S.: 10–100 mm	Methanol (10–100% v/v)	P: 20–50% of 700 W TOE: 10–20 min S.C.: 10–60 mL/g	Y: 45.61 mg of TFC/g O.C.: 15 min, 40 mL/g, 210 W	[160]
*Apium graveolens* L. (celery) M: 2 g P.S.: —	Ethanol (50–80%)	P: 300–500 W TOE: — S.C.: 10–30 mL/g of solvent-to-solid ratio	Y: — O.C.: 30 mL/g, 500 W, 75.6% (v/v)	[161]
*Periploca forrestii* Schltr. M: 1 g P.S.: 60-mesh sieve	Ethanol (50–70%)	P: 210–350 W TOE: 180–240 s S.C.: 15–25 mL/g, microwave combined with ultrasonic-assisted extraction	Y: 19.86% O.C.: 209 s, 21.24 mL/g, 274 W	[162]

^a^M: mass; P.S: particle size (average) or sieved (no. of meshes). ^b^Extraction without solvent (none) using natural moisture. ^c^P: power of MW; TOE: time of extraction; S.C.: special conditions. ^d^Y: yield (expressed as QE: quercetin equivalent; TFC: total flavonoid content); O.C.: optimum conditions (time of extraction, solvent-to-solid ratio, energy, power, temperature, solvent concentration, and particle size).

**Table 6:** Experimental conditions for enzyme-assisted extraction of flavonoids.

Source	Experimental conditions	Flavonoid	Enzyme	Yield	Reference
Guava leaves	pH: 5.0 Temp: 50°C Enzyme: substrate: 0.5/5 g/g Enzyme mixture: substrate: 1.5/5 g/g Time: 12 h Solv: water	Quercetin	Cellulase Xylanase *β*-glucosidase Mixture cellulase: xylanase:*β*-glucosidase (1 : 1 : 1)	106.7 ± 1.21 mg/100 g 72.8 ± 1.36 mg/100 g 199.0 ± 1.36 mg/7100 g 258.9 ± 3.32 mg/100 g	[222]
Pomegranate peels	pH: — Temp: 44.85°C Enzyme: substrate: — Enzyme mixture: substrate: — Time: 41.45 min Solv: water	TFC	Viscozyme® (1.32 mL/100 mL)	17.97 mg/g	[223]
*Momordica balsamina* L. fruit	pH: 7.5 Temp: 50°C Enzyme: substrate: 6.5% Enzyme mixture: substrate: — Time: 60 min Solv: 80% MeOH	TFC	Zympex-014®	Approx. 18 mg/g	[224]
*Citrus sinensis* peel	pH: 4.8 Temp: 60°C Enzyme: substrate: 30.94 mL/g Enzyme mixture: Substrate: — Time: 4.87 Solv: —	TFC	Viscozyme L®	264.6 mg/100 g	[225]
*Medicago sativa* leaves	pH: 5.8 Temp: 45°C Enzyme: substrate: 2.9% Enzyme mixture: substrate: — Time: 90 min Solv: —	TFC	Kemzyme®	62.55 ± 2.43 *μ*g/g	[226]
*Tagetes erecta* L. flower	pH: — Temp: 45°C Enzyme: substrate: 0.45 U/g Enzyme mixture: substrate: — Time: 150 min Solv: 20% EtOH	Luteolin	Pectinase	7.32 mg/g	[227]
Chokeberry pomace	pH: 3.5 Temp: 40°C Enzyme: substrate: 6% v/w Enzyme mixture: substrate: — Time: 7 h Solv: —	Dihydroquercetin	Viscozyme L®	8.01 mg/g (after SFE) 1.00 mg/g (after PLE)	[228]
*Laurus nobilis* L. leaves	pH: — Temp: 40°C Enzyme: substrate: 1 mg/10 g Enzyme mixture: substrate: (1 : 1 : 1) Time: 1 h Solv: MeOH	TFC	Cellulase Hemicellulase Xylanase Ternary mixture	5.79 ± 0.41 mg/g 5.35 ± 0.22 mg/g 6.33 ± 0.38 mg/g 6.09 ± 0.78 mg/g	[229]

Solv: solvent, Temp: temperature, TFC: total flavonoid content, EtOH: ethanol, MeOH: methanol, U: unit of enzymatic activity, SFE: supercritical fluid extraction, and PLE: pressurized liquid extraction.
